# Community Dynamics Drive Calcium Carbonate Production in an Enriched Consortium of Soil Microbes

**DOI:** 10.1007/s00248-025-02632-y

**Published:** 2025-12-23

**Authors:** Marci Garcia, Natalie C. Sadler, Izabel Stohel, Sharon Zhao, Sankarganesh Krishnamoorthy, Yuliya Farris, Nicholas J. Reichart, Christopher E. Bagwell, Neerja Zambare, Ryan McClure

**Affiliations:** 1 Biological Sciences Division, Pacific Northwest National Laboratory, Richland, WA USA; 2 Environmental Molecular Sciences Division, Pacific Northwest National Laboratory, Richland, WA 99352 USA

**Keywords:** Soil microbiome, Carbon sequestration, Carbon storage, Calcium carbonate, MICP, Model community, Model consortium

## Abstract

Recently, there has been a focus on using soil microbes as a means to store carbon in the soil in the form of calcium carbonate, outcomes of which include soil stabilization and biocementation. The molecular processes involved in microbially induced calcium carbonate formation are known, but there is still a significant knowledge gap regarding how community interactions, emergent processes that are distinct from the roles of individual members, may drive the formation of carbonate. To answer these questions, we describe the development and application of a consortium of soil microbes consisting of one species each of the *Rhodococcus*, *Microbacterium*, and *Curtobacterium* genera and two species from the *Bacillus* genus. We term these five species cultivated together carbon storing consortium A (CSC-A). Growth assays show that only a subset of CSC-A members produces CaCO_3_ with *Rhodococcus* producing the most CaCO_3_ but the complete CSC-A produces significantly higher amounts of CaCO_3_ compared to the sum total carbonate produced by all member species. The development of CSC-A shows that CaCO_3_ production may be as much a community process as it is the contribution of individual species, requiring us to move beyond single species analysis to fully understand carbonate formation by microbial communities in nature. CSC-A will allow the scientific community to ask and answer key questions about the molecular interactions surrounding inorganic carbon formation in soil, an important knowledge gap that must be filled if we wish to stabilize soils and harness microbial processes for materials production.

**Supplementary Information:**

The online version contains supplementary material available at 10.1007/s00248-025-02632-y.

## Introduction

Much of the focus of soil science is targeted to nutrient cycling, especially cycling of carbon in the soil. To that end, much effort has gone into examining organic forms of carbon in soil (plant roots, microbial biomass, etc.), but soil inorganic carbon (SIC) also plays a major role in the soil ecosystem. Inorganic forms of carbon, such as carbonates, can lead to more stable soils, especially those with higher clay content [1, 2]. Carbonate production has also been shown to be an effective method to bioremediate soils containing heavy metal contamination [3]. In addition, soils represent an important carbon sink with the global soil ecosystem able to store up to 2500 gigatons of carbon [4]. This represents more than 80% of the terrestrial soil carbon, far more than is found in plant biomatter. However, there are significant knowledge gaps that must be filled before we can take real steps to capture inorganic carbon in soil [5]. Moving CO_2_ from the atmosphere to a soil sink is primarily done through photosynthesis via plant growth and then transferring this carbon belowground as root exudates or plant litter. Plant litter and root exudates are then used as nutrients by soil microbes [6]. In some cases, specific root exudates are produced by plants to recruit certain microbial species to the root zone (the rhizosphere) to help the plant respond to certain stress conditions such as drought or fungal pathogens [7]. Depending on environmental conditions during microbial metabolism of root exudates and other carbon sources, certain fractions of carbon are routed to increased microbial biomass or are respired back into the atmosphere as CO_2_. Aside from these routes, other pathways exist for soil carbon, one of which is the production of carbonates (anions) by microbial species [8]. The carbonate molecules can then combine with cations, such as calcium, in the soil, to produce calcium carbonate, CaCO_3_, among other molecular species [9]. CaCO_3_ (calcite) is one of the most abundant forms of carbon in the soil, especially in arid or semi-arid regions, while other carbonate species, such as dolomite, are more common in sediments [10]. Once in this form, carbon is much less labile and soluble, making it harder to use as inputs for further microbial metabolism, though it can be released via acidification as a result of microbial or human activity [11]. This stability means that such carbon can remain in the soil and provide benefits (prolonged stable sequestration, soil stability, etc.) for the long term.

One of the most energy efficient and common ways in which microbially induced calcium carbonate precipitation (MICP) is carried out is through urea hydrolysis, or ureolysis, via urease enzymes [12, 13]. Microbial ureolysis produces ammonia and CO_3_^−2^; the former increases the local pH which in turn creates conditions that allow the latter to bind to available cations, such as Ca^2+^, to form insoluble CaCO_3_ precipitates [14]. A number of soil microbes have been found that can carry out this process including, but not limited to, *Sporosarcina pasteurii* [15], *Pseudomonas* sp. [16], *Variovorax* sp. [17], *Micrococcus luteus* [18], and *Bacillus cereus* [19]. An additional way in which production of CaCO_3_ can be driven by microbes is through expression of carbonic anhydrase enzymes [20]. Identified carbonic anhydrase producing soil microbes include *Virgibacillus* [21], *Ralstonia* [22], and *Pseudomonas* [23]*.* Carbonic anhydrases may be of even greater interest as their activity consumes CO_2_, rather than urea, to generate carbonate, further reducing total CO_2_ levels.

Many of the specific species, as well as the molecular processes and enzymes, that are involved in MICP are well known [12]. However, within the native soil ecosystem, no species exists in isolation. The production of CaCO_3_ by the soil microbiome is an outcome of the complete soil microbial community, not only individual species but also the interactions they have with other members. These species may include those that do not produce CaCO_3_ themselves but who may have critical roles at the community level that enhance the production of CaCO_3_ in the soil as a whole. Many additional processes carried out by the soil microbial community have been found to be driven by the interaction network of these microbial species (e.g., nutrient decomposition or nitrogen fixation) [24, 25]. The same is likely true for communities that mineralize carbon. Because CaCO_3_ production is likely as much a community outcome as it is a process driven by individual species, it is critical for us to understand these community processes if we want to harness the soil microbiome to drive the formation of inorganic forms of carbon.

Despite the need to understand interactions within soil microbiomes related to MICP, this is often a difficult goal to reach. The soil microbial community is incredibly complex, and direct analysis of this ecological system to fill knowledge gaps is difficult. Recently, success has been seen in reducing the complexity of the soil microbiome into model, representative communities that contain fewer species and are more amenable to -omics assays and other studies designed to better understand the molecular basis of interactions and how they lead to expressed functions [26–28]. While caution should be applied to any discoveries made from analysis of a consortium with regard to their relevance to natural, more complex, systems, the development and study of such consortia can lead to new, testable hypotheses and fundamental paradigms that can then be tested in the complete and natural system. To that end, the development of a defined, reduced complexity, soil-derived microbial consortium that can carry out MICP would be a major step forward in harnessing soil systems for carbon storage. Here, we describe the development and analysis of a carbon storing consortium (CSC) comprised of soil bacteria and show that such a consortium can drive the formation of large amounts of CaCO_3_ as a result of synergistic interactions between members.

## Methods

### Media Formulations and Species Growth

*S. pasteurii* ATCC #11,859, *Bacillus subtilis* ATCC #6051, and *Escherichia coli* MJK2 (received from collaborators at Montana State University [29]) were used to test various media compositions to determine the best nutrient environment for CaCO_3_ formation. *S. pasteurii* and *B. subtilis* are well-characterized urease producers, while the *E. coli* strain, MJK2, was previously transformed to carry a pJN105 plasmid modified to include a urease operon under the control of a l-arabinose-inducible promoter which encodes for gentamycin resistance [29]. To start any species for MICP analysis (either the three test species above or constituents of the developed CSC-A, described below), each species was plated out from freezer stocks onto agar plates. For *S. pasteurii*, BHI medium was used; for *E. coli* MJK2 and *B. subtilis*, LB medium was used. For all CSC-A constituents in later experiments, R2A medium was used. Species were grown on agar plates for 1–2 days at 20 °C. Following initial growth, species were reinoculated into one of several different media types depending on the goals of the experiment (Supplementary Table 1) aiming for a starting O.D._600 nm_ of 0.1 (determined via a Nanodrop spectrophotometer, Thermo Fisher). The pH of each liquid medium was adjusted to 6.0 before being filter-sterilized with a 0.22-µm PES vacuum-driven filtration device (Biologix, Camarillo, CA). To make agar plates, a 2 × solution of each medium was made and was used to dilute an identical volume of a 3% agar solution for a final set of plates with 1 × of each medium type and 1.5% agar. All growth assays took place at 20 °C, with liquid assays also shaken at 150 rpm in a New Brunswick 2180 shaker. For experiments that required a pH measurement, 0.0012% phenol red was added to liquid media or plates as a pH indicator.

### Analysis of CaCO_3_ Precipitate

To collect and quantify CaCO_3_ precipitate, we inoculated individual strains or the complete CSC-A at a starting OD of 0.1 in 100 mL of medium in flasks. At certain timepoints defined by the experiment, a 30-mL aliquot was removed. The liquid aliquots were allowed to sit for 30 min at room temperature leading to the settling of CaCO_3_ at the bottom of the container. The liquid portion was removed by decanting and discarded. CaCO_3_ was then washed with deionized water (using 1/10 the volume of the liquid removed from the container) and then spun down at 5000 g for 5 min. This wash step was repeated once, and clean CaCO_3_ was stored at − 80 °C until quantification. For quantification, CaCO_3_ was resuspended in 800 µL of water and placed in a pre-weighed aluminum boat for weighing. Boats were heated at 100 °C overnight to evaporate water and weighed again. Boats were then heated at 550 °C for 30 min to burn off any residual biomass and weighed a final time. Fluorescence microscopy was used to visualize CaCO_3_ precipitates within growing cultures utilizing the innate auto fluorescence of CaCO_3_. To do so, 100 µL of culture from each sample was first treated with 1X SYBR™ Gold Nucleic Acid to stain the microbial cells, incubated in the dark for 20 min, and then loaded into a 12-well imaging chamber (Ibidi) attached to #1.5 glass coverslips. An inverted confocal microscope (Dmi6000b, Leica Microsystems, Wetzlar, Germany) equipped with a CSU 10 confocal scanning unit (Yokogawa Corporation of America, Sugar Land, TX) was used for this work. The instrument was fitted with a Leica Plan APO 20/0.7 objective that uses CoolSNAP HQ2 (Photometrics, Tucson, AZ, USA) controlled by MetaMorph version 7.7.8.0 (Molecular Devices, Sunnyvale, CA, USA) software. Laser line 405 nm Ex; 460/50 nm Em was used for detecting CaCO_3_ autofluorescence and laser line 488 nm Ex; 525/50 nm Em was used for detecting SYBR™ Gold-stained cells. Images for both wavelengths were combined and processed in ImageJ 1.53a. For X-ray diffraction (XRD), each sample was prepared by crushing the sample into fine powder using a mortar and pestle. Diffraction data were collected from 3 to 90° 2*θ*, scanning at 0.5° min^−1^ and recording counts every 0.04° (5 s), using a Rigaku MiniFlex II Bragg–Brentano diffractometer with Cu-Kα radiation (*λ* = 1.5418 Å) and a graphite post-diffraction monochromator. Identification of material phases was carried out using JADE® and TOPAS® XRD pattern processing software and reference patterns from the International Centre for Diffraction Data (ICDD) powder diffraction database.

### Development of a Calcium Carbonate Producing Consortium

Soil samples were collected from the Kearney Agricultural Research and Extension (KARE) Center in Parlier, California, and shipped to the PNNL campus in Richland, Washington. A total of 1 g of soil was diluted into 9 mL of B4 medium, representing a 10^−1^ dilution. One mL of this dilution was then added to 9 mL of B4 medium (a 10^−2^ dilution), and 1 mL of this 10^−2^ dilution was then added to 9 mL of B4 for a 10^−3^ dilution. Three replicates of each dilution were made. A total of 500 µL of each of the three 10^−3^ replicate dilutions was then spread onto three separate B4 agar plates containing 0.0012% phenol red. This process was repeated with 10^−3^ dilutions on B4U, B4UNZ, and B4NZ plates containing 0.0012% phenol red. All media compositions are described in Supplementary Table 1. This led to a total of 12 agar plates. Following plating, an additional 1.5 mL of each of the three 10^−3^ replicate dilutions was removed and added to 13.5 mL of B4 medium containing phenol red (the same amount as in the agar plates above). This process was repeated with additional aliquots of the 10^−3^ dilution added to 13.5 mL of B4U, B4UNZ, or B4NZ medium containing phenol red. This led to a total of 12 liquid medium tubes. Agar plates were incubated at 20 °C, and liquid medium was incubated at 20 °C with shaking as above. Every 3–4 days, plates and liquid growth were passaged, and samples were collected for DNA extraction. For passaging agar plates, all growth was collected with a cell scraper and resuspended in 1.4 mL of sterile PBS. From this resuspension, 100 µL was spread onto a new agar plate (of the same medium type that the growth was collected from). The remaining 1.3 mL was spun down at 5000 g for 5 min with the pellet then stored at − 80 °C for DNA extraction. For liquid medium, 500 µL was removed from the tubes and added to 14.5 mL of fresh medium (of the same medium type that the growth was collected from). An additional 1.3 mL was collected from each tube and spun down and stored as above for eventual DNA extraction from the resulting cell pellet. This process was continued for nine passages (roughly two passages each week). Photographs were taken at each passaging (Supplementary Figs. 2–8).

### 16S rRNA Amplicon Analysis

DNA was extracted from microbial cell pellets using the ZymoBIOMICS DNA miniprep kit (Zymo Research, Irvine, California). Ten microliters of each sample was transferred to a well of a 96-well plate containing the necessary barcoded forward primer and reagent cocktail mixture. The forward primer (1 µL, 0.2 µM final concentration) targeted the V4 region (5′-GTGYCAGCMGCCGCGGTAA–3′) and contained unique 12 base barcodes. The reagent cocktail mixture contained 20 µL Platinum II Master Mix (Invitrogen; 0.8 × final concentration), 1 µL 806 reverse primer (5′-GGACTACNVGGGTWTCTAAT–3′; 0.2 µM final concentration), and 18 µL water to make a 50 µL final volume reaction. The plates were sealed and loaded onto a ProFlex PCR Systems thermocycler (Applied Biosystems, Waltham, Massachusetts) and run under the following conditions: initial hot start at 74 °C for 3 min, followed by 35 cycles of 94 °C for 60 s, 55 °C for 45 s, and 74 °C for 90 s, before a final extension step at 74 °C for 10 min. After completion of PCR, samples were assayed for DNA concentration using the Quant-iT PicoGreen dsDNA assay kit (Invitrogen) in triplicate reactions. A total of 200 ng per sample was pooled into a single tube, or up to 40 µL if 200 ng could not be achieved and cleaned of excess primers using a Zymo Clean and Concentrator–100 kit (Zymo Research, Irvine, California).

The pooled and cleaned sample was further diluted to 2 nM, and the recommended protocol for Illumina v2 500 cycle chemistry for the MiSeq was followed. PhiX was used at a final 15% concentration and mixed into the sample prior to loading on the MiSeq. Data was exported from the MiSeq as demultiplexed fastq files. Demultiplexed reads were processed using Qiime2 (version 2021.4) [30]. The adapter sequences were trimmed, and reads were truncated to maintain quality using DADA2 [31] (forward, 185 bp; reverse, 150 bp). Taxonomy was assigned as amplicon sequence variants (ASVs) using the Silva SSU database release 138 with the classify-sklearn plugin within the Qiime2 environment. Further read processing occurred using the R programming language. The package decontam [32] was run to remove ASVs identified as contaminants using a 0.5 threshold with the Prevalence model. Samples containing less than 5000 reads were discarded, as were singleton ASVs. The remaining reads were analyzed using Phyloseq [33], vegan [34], and DESeq2 [35] packages for diversity metrics and statistical tests with ggplot for figure generation. Principal component analysis was done in R using the prcomp function, part of the R stats package [36]. PCA plots were made using the autoplot function, part of the R ggbio package [37].

To identify species isolated from the consortium, we used the 16S rRNA primers listed above and sent the resulting PCR product for Sanger sequencing using Functional Biosciences (Madison, WI).

## Results

### Identifying a Medium Promoting Calcium Carbonate Formation

Our ultimate goal was the development of a consortium of soil bacteria that is enriched for a CaCO_3_ formation phenotype. The choice of medium used to evolve this consortium will have a large effect on which species are retained and which are competed out. To identify which media source might be best for our desired goals, we tested five different media types against three species of bacteria known for their potential to drive CaCO_3_ production: *S. pasteurii* ATCC 11859, *E. coli* MJK2, and *B. subtilis* ATCC 6051*.* All three species grown in B4U and B4UNZ medium induced large amounts of CaCO_3_ formation (Supplemental Fig. 1). B4U medium led to CaCO_3_ levels that were between 2.7-fold and 34-fold higher than CaCO_3_ amounts with either NBU, NBUNZ, or NZECM levels in either *E. coli* or *B. subtilis*. B4UNZ medium led to CaCO_3_ levels that were between 3.5-fold and 26-fold higher than CaCO_3_ amounts with either NBU, NBUNZ, or NZECM levels in either *E. coli* or *B. subtilis*. NZECM did lead to very high levels of CaCO_3_ in *S. pasteurii*. However, the goal of this project was to enrich for a diverse set of MICP bacteria, and since NZECM seemed to lead to CaCO_3_ formation only in one species out of the three tested, its effects might not be applicable to a wide variety of bacterial species and it was set aside. With these results, we chose to move forward with consortia development using B4U and B4UNZ media. Since media containing urea will tend to enrich for a community containing ureolysis as a pathway to drive carbonate production, we also wanted to explore the effect of other media types. We therefore also chose to develop consortia on the B4U and B4UNZ media lacking urea (B4, B4NZ).

**Fig. 1:**
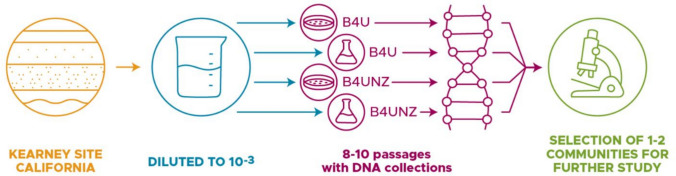
Schematic of experimental approach. Soil was collected from a field site in California and shipped to Washington state. Soil was diluted and plated on agar plates or used to inoculate liquid media. Every 3–4 days, emerging microbial growth was passaged. After 8–10 passages, 1–2 communities were selected for further analysis

### Enriched Communities of CaCO_3_-Producing Species

**Fig. 2:**
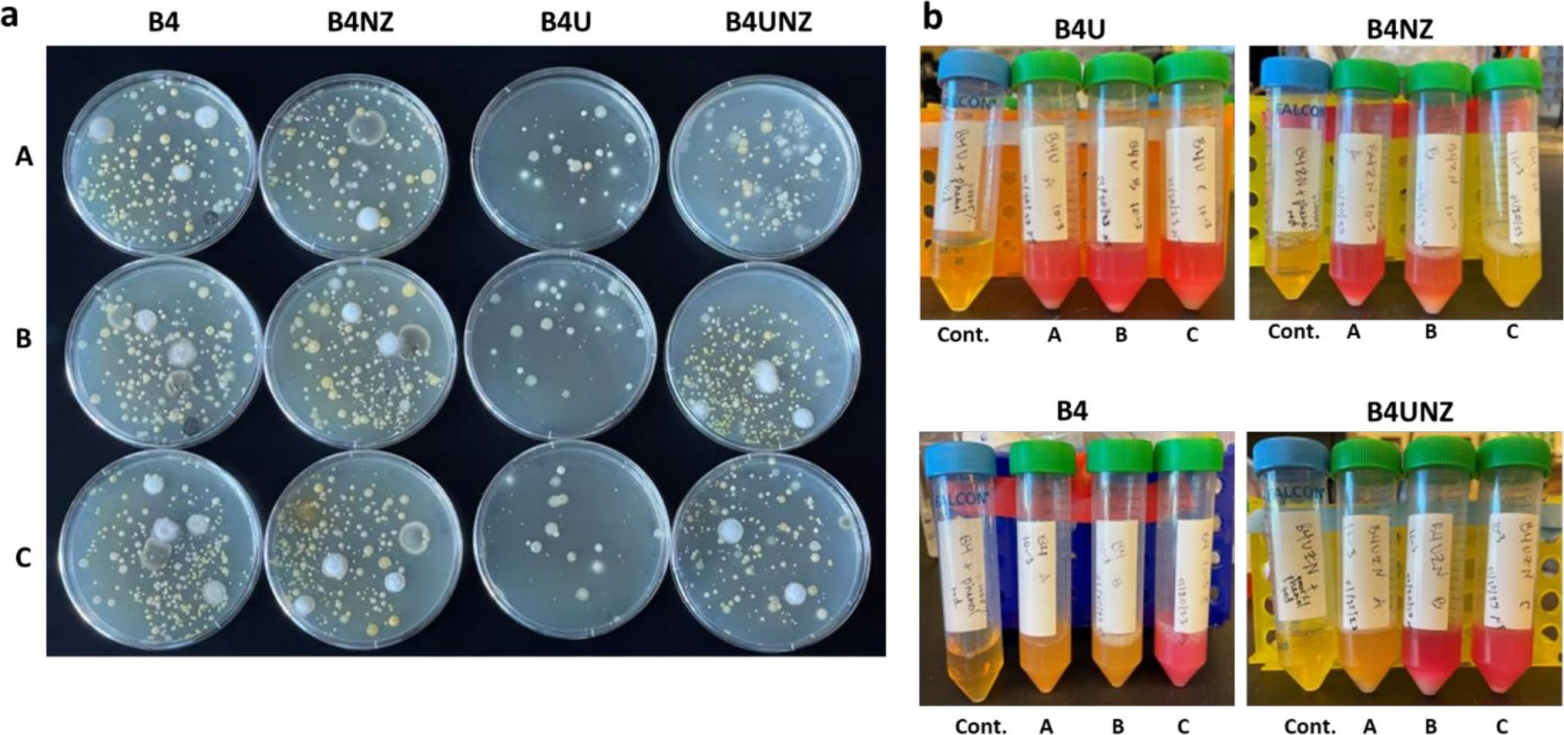
Early stages of community development. **a** Photos of R2A agar plates after 1 week of growth following spreading of diluted soil. Letters on the horizontal indicate replicates. Labels on the vertical indicate media types (Supplemental Table 1). **b** Photos of liquid media after 1 week of growth following inoculation of soil dilution. Media types are indicated on the top of each picture and inoculated replicates on the bottom. “Cont.” indicates the uninoculated control

**Fig. 3:**
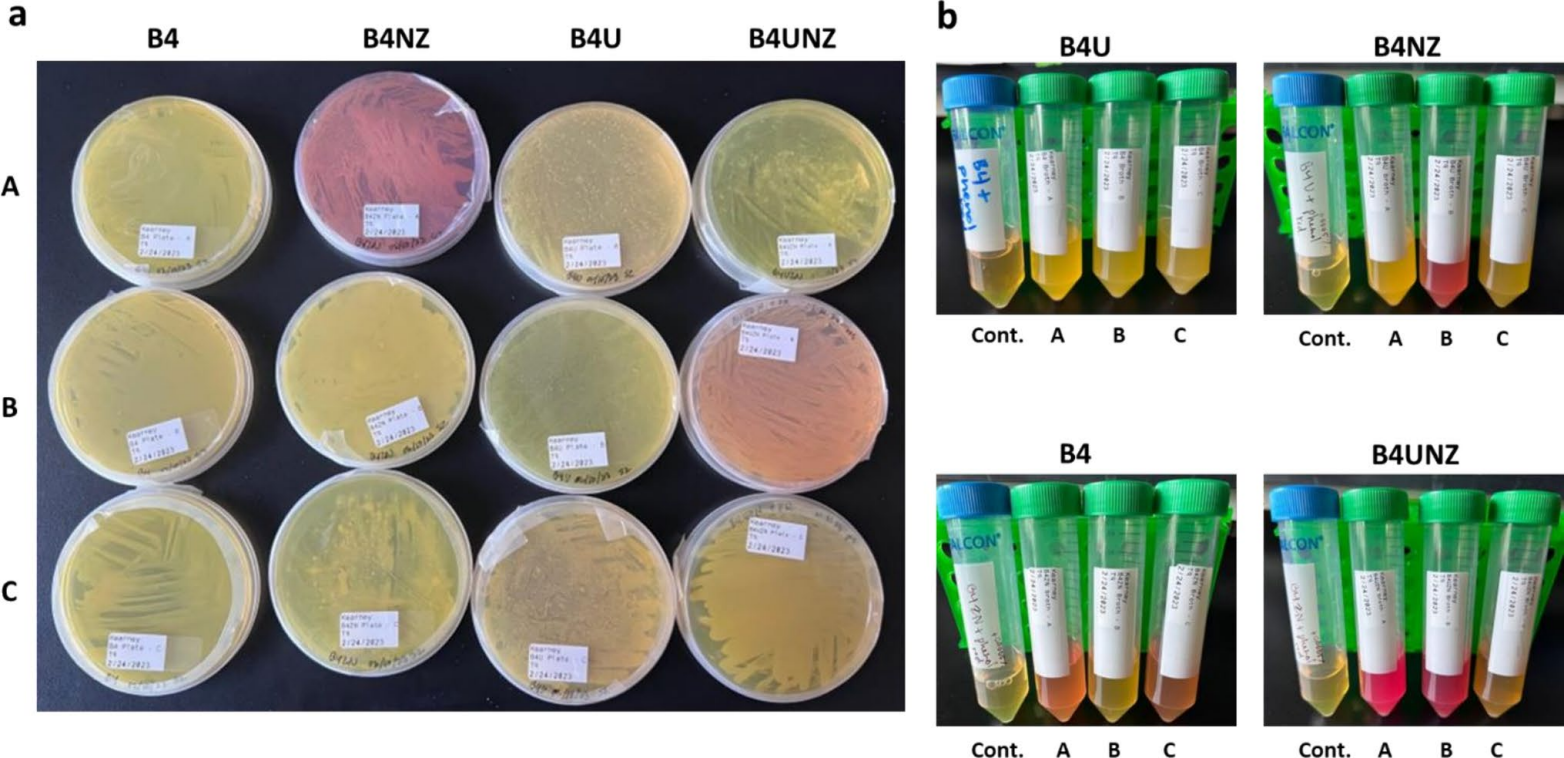
Final stages of community development. **a** Photos of R2A agar plates after nine passages. Letters on the horizontal indicate replicates. Labels on the vertical indicate media types (Supplemental Table 1). **b** Photos of liquid media after nine passages. Media types are indicated on the top of each picture and inoculated replicates on the bottom. “Cont.” indicates the uninoculated control

We chose to use both agar plate assays and liquid growth assays to evolve our communities (Fig. 1). While this approach obviously selects only for lab-culturable species and would tend to select for fast-growing species, this was part of our design. We will need a final consortium of culturable species if we wish to better understand the details of species interactions that drive CaCO_3_ formation. A faster-growing consortium also lends itself better to laboratory work. We also included a phenol red indicator in subsequent platings after an initial week of growth to track changes in pH as carbonate precipitation increases as the environment becomes more alkaline. Our initial week of growth after agar plating showed a wide variety of isolate colonies; of all media types, B4U medium showed the fewest number of colonies (Fig. 2A). Growth in liquid media was robust, and several replicate communities showed shifts to a red color (indicating higher pH) (Fig. 2B). Passaging of plates and liquid growth continued every 3–4 days. In general, communities grown in liquid culture increased the media pH over time (comparing 12-h timepoints to 72-h timepoints, data not shown). Notably, there did not seem to be a strong correlation between replicate tubes of the same media type when looking at either increases in pH or white precipitate (hypothesized and later confirmed to be CaCO_3_) suggesting a large amount of stochasticity in the evolution and expressed phenotypes of replicate communities. This was later supported by 16S rRNA analysis showing that in many cases, communities started with similar taxonomic profiles and then diverged with time. It is also possible that with more replicates—greater than the three we have here—a statistical link would emerge between starting tubes and pH changes or the development of white precipitate. Communities that are slower-growing will lead to less precipitate and pH increases, even if they have the genomic and phenotypic potential to drive CaCO_3_ production. Using B4U medium, we initially saw a consistent increase in pH in Replicates A, B and C, but after week 5, this decreased and subsequent replicate communities with this medium type did not show pH changes or white precipitate. For communities made using B4NZ medium after week 2, Replicate B showed consistent changes in pH; however, white precipitate was not visible in this consortium. For consortia developed using B4 medium, we saw consistent pH changes in Replicate B as well as large amounts of white precipitate but this faded by the end of the development. Finally, use of B4UNZ medium showed some variability between replicates, but by the last few weeks, consistent pH changes were noted in Replicates A and B with white precipitate also being observed in both tubes (though less than that with B4U medium) (Fig. 3B). Looking at consortia developed on plates, we did see more consistent responses across medium types but still some variability between replicates. Generally, Replicate A from B4NZ medium show consistent increases in pH (color change on plates from yellow to red). A similar trend was also seen with Replicates B and C using B4UNZ medium though this did have some variability (Fig. 3A). In some cases, white crystals could be seen on the surface of the microbial lawn, but this was more difficult to quantify and verify compared to the white precipitate seen in liquid medium. Photographs of all plate and liquid growth of communities after each passage are shown in Supplementary Figs. 2–8.

### Selection of Species for a Carbon Storing Consortium

**Fig. 4:**
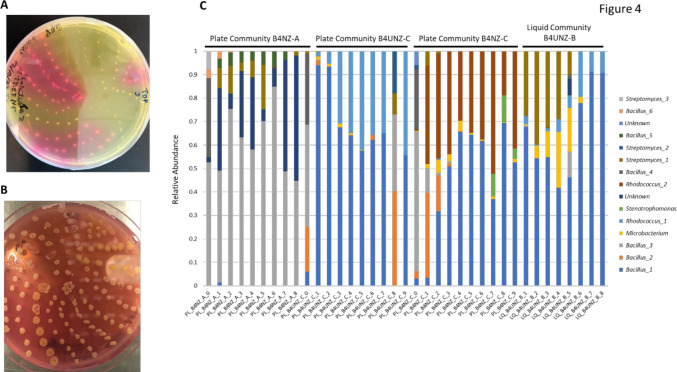
Community isolates and 16 s analysis. **A** B4UNZ agar plate with pH indicator and several isolates from evolved communities. Areas of the plate that are pink indicate a local rise in pH. **B** The same plate as in (**A**) but after several more days of growth. Crystalized CaCO_3_ is visible on the tops of several colonies. **C** 16S rRNA amplicon data. Relative abundances are shown on the y-axis. Communities are indicated along the x-axis, and community groups are indicated above. Taxonomies are indicated by color. “Bacillus_3” and “Bacillus_4” for example indicate *Bacillus* genera that could be differentiated by 16s rRNA sequencing but for which the species identity is not known

At the conclusion of consortia development, we selected four consortia that appeared to drive the production of the largest amounts of CaCO_3_ based on visual examination of tubes and plates. These were plate consortium B4NZ-A, plate consortium B4NZ-C, plate consortium B4UNZ-C, and liquid consortium B4UNZ-B. Each of these consortia was then diluted to between 10^−1^ and 10^−3^ using R2A medium and plated on R2A agar plates to isolate individual colonies and eventually constituent species. Constituent selection took place after the development of consortia so that we would be more likely to select isolates that would combine to form a stable community. After 1 week, individual colonies began to emerge. These were collected and replated onto B4UNZ agar plates with phenol red. As we wanted to develop a consortium comprised of species that may drive the production of CaCO_3_ and those that may not (to better understand interactions between species with different phenotypes), we selected colonies that led to colorimetric changes in the agar plates (indicating increases in pH due to added phenol red) and those that did not (Fig. 4A). This fulfilled our goal of a consortium that would likely contain CaCO_3_-forming members as well as members not driving the formation of CaCO_3_. We also selected those that led to crystals forming on the tops of the colonies (Fig. 4B) (likely CaCO_3_ crystals). The variability in color changes on the plates as a function of CFUs was our first indication that not all of the species being isolated from these consortia have a CaCO_3_ production phenotype. Selected isolates were then passaged at least three more times on R2A agar before being collected for DNA extraction and 16S rRNA Sanger sequencing. We eventually chose 51 isolates to send out for sequencing. In some cases, 16S rRNA Sanger sequencing was repeated twice to ensure correct identification. We also tested each isolate in liquid culture to determine if pH levels rose, an indication that the isolate may drive the production of CaCO_3_ (SupplementaryTable 2).

16S rRNA sequencing consistently determined many of the same genera and, in some cases, species of the isolates. This observation was also supported by our 16S rRNA amplicon analysis of all consortia (liquid, plate, medium type, and replicate, Fig. 4C). Amplicon analysis showed that many of the same species emerge as consortia evolve, but there are some taxonomic differences between consortia and major differences in the abundance of species common to more than one consortium depending on the medium type, environment (liquid or plate), and even the replicate. Plate consortium B4NZ-A was comprised almost exclusively of several *Bacilli* species with some *Streptomyces* membership as well. In contrast, plate consortium B4NZ-C was comprised of separate *Bacilli* species as well as very high levels of *Rhodococcus* species and a small amount of *Stenotrophomonas*. The amplicon analysis also showed the importance of sufficient passaging to allow for the greatest amount of consortium stability. With each passage, the consortia generally changed less compared to the previous passage showing that sufficient passaging can lead to a more stable community. Even in the last few passages, however, some consortia were still changing, though more slowly than at the beginning of the evolution. 16S analysis also provided a closer view on how stochasticity may be a factor in consortium development. PCA plots showed how some environments (plate or liquid) and medium combinations contained consortia that were very similar taxonomically at the start of consortia development but then diverged (Supplementary Figs. 9 and 10). For example, in consortia developed in liquid medium with either B4 or B4NZ medium, replicates of a given starting consortia and early passages (Week 0 and Week 1) were similar, but as passaging continued (Weeks 4–8), replicate consortia became taxonomically more and more divergent, showing that even consortia that start off very similar can rapidly diverge even under the same cultivation conditions. This was also the case with replicate consortia in B4 and B4NZ medium developed on agar plates. However, for consortia developed using B4U or B4UNZ medium (in either liquid medium or on plates), starting consortia were much more diverse, suggesting that initial dilutions of the soil may have led to differences in the start point rather than stochasticity as the consortium developed. 16S rRNA sequencing data is shown for all consortia in Supplementary Figs. 11 and 12 and also supports the observation that vastly different consortia arise in liquid or plate environments.

The five taxa that were most commonly found during our isolation of species were *Bacillus thuringiensis* (isolate 52 in Supplementary Table 2), *Bacillus toyonensis* (isolate 43), *Curtobacterium flaccumfaciens* (isolate 9), *Rhodococcus qingshengii* (isolate 40), and a *Microbacterium* sp. (isolate 31). In the case of the two *Bacillus* sp., the *Rhodococcus* and the *Microbacterium*, the species were often found in the same consortium. The *Curtobacterium* species was only isolated from a single consortium, one grown on agar plates using B4ZN-A medium. Based on the variability in the pH and carbonate-producing effect of the five most common species, as well as their consistent co-emergence from a complex soil community, we chose to include all five species in our test consortium examining CaCO_3_ production. For *Bacillus thuringiensis*, *Bacillus toyonensis*, *Rhodococcus qingshengii*, and the *Microbacterium* sp., these members were isolated from the same developed consortium (Lq-B4UZN-B in Supplementary Table 2). The *Curtobacterium* member was isolated from Pl-B4ZN-A. All of these are known soil species, not commonly found in humans. We term this combined test consortium carbon storing consortium A (CSC-A).

### Growth and pH Shifts of Member Species of CSC-A

**Fig. 5:**
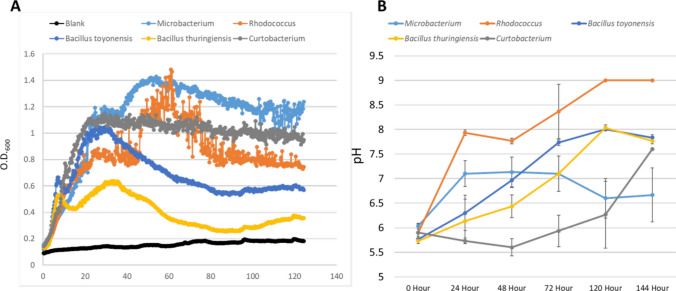
Growth and pH shifts of CSC-A members. **A**. Growth curves of CSC-A members (indicated by color). Optical density at 600 nm is on the y-axis, and hours are on the x-axis. **B** pH shifts of medium during cultivation of CSC-A members. pH is on the y-axis, and hours are on the x-axis. Error bars indicate standard deviation

We next tested the growth of each of the five member strains of CSC-A on B4UNZ medium (the medium which led to the greatest CaCO_3_ for *E. coli* and *B. subtilis*, Supplementary Fig. 1, and contains Nickel, an important cofactor for urease enzymes [38]). Growth rates of each species can be used as input to begin to hypothesize interactions and faster growing species that also drive CaCO_3_ production will be a larger factor in CSC-A carbonate production rates. *Microbacterium* showed initial fast growth and had the highest final O.D. The two *Bacillus* strains (*B. toyonensis* and *B. thuringiensis*) showed similar growth profiles over time though *B. toyonensis* did have a higher O.D. *Curtobacterium* rapidly increased to a final O.D. of ~ 1.2 where it remained with a long, stable stationary phase. *Rhodococcus* showed the slowest growth increase but peaked briefly at the highest O.D. Data from *Rhodococcus* was also quite noisy suggesting significant cell clumping (Fig. 5A). We also measured the role each species has in changing pH as higher pH levels would lead to a more amenable environment for the formation of CaCO_3_. In most cases, pH changes by individual species were very different than O.D. changes. B4UNZ medium cultured with *Rhodococcus* showed a rapid increase in pH, reaching ~ 9.0. *Microbacterium* also showed a rapid increase, but the final pH was only slightly above 7.0. Both *Bacillus* strains showed a slower increase in pH ending between *Rhodococcus* and *Microbacterium* (pH of ~ 7.75). *Curtobacterium* showed the slowest change; pH levels did not begin to rise until roughly 72 h after the start of the experiment, but by 144 h, the pH change was rapidly increasing and had reached greater than 7.5 (Fig. 5B).

### CaCO_3_ Production by Each Member Species of CSC-A and the Full Consortium

We next grew each member species as well as the combined CSC-A using B4UNZ medium and measured the amount of CaCO_3_ that precipitated with each strain and in CSC-A. We first took additional steps to confirm that the white precipitate we were seeing in later stages of consortium development was in fact CaCO_3_. We used microscopy to confirm that the precipitate exhibits UV excitable autofluorescence, a known characteristic of some polyforms of CaCO_3_ precipitates (Supplemental Fig. 13) [39, 40]. In addition, the white precipitate only appears when the pH becomes more basic allowing for the carbonate to fall out of solution; we confirmed that this precipitate goes back into solution when the pH is lowered by adding acid to the sample (Supplementary Fig. 14). Finally, X-ray diffraction analysis confirmed that 75% of the sample is vaterite, 5% is calcite, and the remaining 20% is amorphous (Supplemental Fig. 15).

**Fig. 6:**
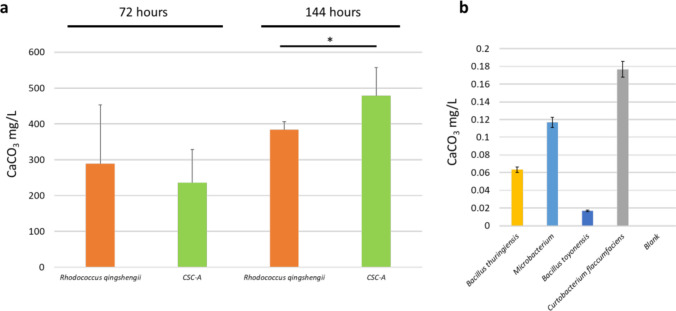
CaCO_3_ amounts produced by CSC-A constituents and the community as a whole.** a** The amount of CaCO_3_ is shown in the y-axis. *R. qingshengii* (a major carbonate producer) and the CSC-A community are shown on the x-axis. Two timepoints were analyzed, indicated on the top of the figure. Asterisk (*) indicates a *p*-value of less than 0.05. **b** A zoomed in view of some of the constituents of CSC-A that produce small amounts of CaCO3 at the 144-h timepoint

We cultured each of the strains independently as well as the complete consortium together and collected CaCO_3_ samples at 72 h and 144 h after the start of the experiment (Fig. 6A). At 72 h after the start of the experiment, CSC-A was driving the production of large amounts of CaCO_3_, ~ 230 mg/L of medium. Among the five strains, only *Rhodococcus* was driving the production of a similar amount of CaCO_3_, ~ 280 mg/L; the remaining four members were led to the precipitation of almost no CaCO_3_, and there was none in a non-inoculated control tube as well. By 144 h, things were very much the same except higher amounts of CaCO_3_ were found with *Rhodococcus* (~ 375 mg/L) and CSC-A (~ 490 mg/L). At this point, we also noted that CSC-A was producing significantly more CaCO_3_ than *Rhodococcus* alone. At the 144-h timepoint, we took a closer look at any CaCO_3_ produced by other members of the community aside from *Rhodococcus* (Fig. 6B). Again, only small amounts precipitated but the amounts were higher than the blank uninoculated control with *Curtobacterium* driving the production of the highest amount among the four followed by *Microbacterium* and the two *Bacilli* strains. For each of the three replicate experiments, the sum of the total CaCO_3_ produced by all member species grown in monoculture was 366.27, 348.39, and 398.38 mg, while the amount produced by the CSC-A community as a whole was 466.28, 543.45, and 467.16 mg, an average increase of 32% (*p*-value of 0.014). The fact that CSC-A produces more CaCO_3_ than the sum of the individual member species at the later timepoint strongly suggests that it is interspecies interactions, not just contributions of species considered in isolation, that lead to the carbon sequestering phenotype of CSC-A.

We next wanted to investigate the taxonomic makeup and stability of CSC-A. We wanted to determine if this consortium was relatively even or was primarily composed of *Rhodococcus*, the only species that drove CaCO_3_ production in monoculture. We found that when all members were combined, the results led to a fairly even consortium, except for *Microbacterium*, which started at a much lower level. After 72 h of incubation, the relative species abundance levels of CSC-A were very much the same, suggesting a stable consortium where one member does not overtake the other four, though this may change at 144 h, beyond the time frame of our 16S rRNA work. There were increases in the amount of *Rhodococcus* and corresponding decreases in *Bacillus thuringiensis* and *Curtobacterium*, but these differences were slight (Supplementary Fig. 16). Thus, the increased CaCO_3_ we see with the CSC-A community is likely not a result of simply higher relative amounts of *Rhodococcus* but rather something more complex. It is possible that in CSC-A, there may be a greater number of *Rhodococcus* cells overall compared to a *Rhodococcus* monoculture, but if this were the case, it still suggests factors driving faster growth of the community compared to the monoculture and a similar knowledge gap to be filled surrounding interactions.

## Discussion

Here, we describe the development and analysis of a consortium of soil bacterial species which have a range of phenotypes related to calcium carbonate production. We show that constituent species of CSC-A can drive the production of CaCO_3_ but that production by the combined growth of all members (CSC-A) exceeds the sum of CaCO_3_ found with individual species. We also show that this consortium is stable across time and that no one species outcompetes the others.

Prior work has identified some individual species and some of the enzymatic processes that are at the center of MICP in soil. However, no species that carries out MICP exists in a monoculture in native ecosystems. Metabolic cross talk and interspecies support are major drivers of soil microbiological functions [41, 42]. Because of this, it is equally important to understand how a community of microbial species interacts to carry out carbonate formation as it is to understand the individual species involved. The development of CSC-A allows us to ask and answer questions related to community production of CaCO_3_. Our results, especially Fig. 6**,** clearly show that CSC-A produces a higher amount of carbonate than the sum of the individual species; the whole is greater than the sum of its parts, pointing to interactions within CSC-A being critical to carbonate production by this consortium. There are several possible ways that interspecies interactions could increase CaCO_3_ in CSC-A. Based on the measurements of individual species, we hypothesize that most of these interactions flow through *Rhodococcus*, the major driver of CaCO_3_ production when species are cultivated separately. One possibility is the production of urea by another species that is then passed to *Rhodococcus* for enzymatic processing by urease. *Rhodococcus* members, including *Rhodococcus qingshengii*, the species used in CSC-A, have been found to have urea transporters [43, 44], and while *Rhodococcus* may be able to synthesize its own urea as well, additional urea from other members of the consortium could enhance carbonate production and CaCO_3_ precipitation at the consortium level further. Future studies examining each member of CSC-A individually using a metabolomic approach could delineate which species make urea for community consumption. It is also possible that other metabolites, not related to urea or carbonate production, could define synergistic interactions between species. Our amplicon analysis shows that many of the species have relatively even abundances when co-cultured suggesting positive interactions, or at least the absence of strong competition. If other species can support the growth of *Rhodococcus* in a general way (aside from urea transfer), this could lead to faster growth of *Rhodococcus* and enhanced CaCO_3_ precipitation. Our analysis of CSC-A included only the full consortium, not subset or pairwise comparisons. However, it is possible that the increased CaCO_3_ production we see with CSC-A is not a function of the complete community but only of a specific set of interactions between *Rhodococcus* and another member. Pairwise incubations between all members, but especially *Rhodococcus*, could test this hypothesis. In addition, our planned next steps include gathering species-specific multi-omic data including metatranscriptomics. Knowledge of what specific processes each member expresses during growth in CSC-A will also help us identify pairwise interactions, identifying where CaCO_3_ production requires CSC-A as a whole or just one or two constituent members. It is also possible that species that show little carbonate production in monoculture may increase MICP when cultured within CSC-A. Phenotypes are not static and can shift due to the surrounding environment and taxonomies [45–48]. Further analysis using pairwise combination experiments [47] or metatranscriptomic analysis [27] will be critical in revealing which species produce the most CaCO_3_ within CSC-A.

The conclusions above would not have been possible without the development and analysis of our reduced complexity consortium, CSC-A. Such model consortia have also been useful in exploring other aspects of soil microbiology [26, 28]. Development of such consortia usually proceeds along two separate routes. Either consortia can be made from combinations of individual isolates [49, 50] or they can be made through cultivating field samples in the lab and allowing for the emergence of an interacting set of microbes using passaging and dilution [28, 51]. Consortia comprised of individual isolates have the advantage of greater control and knowledge regarding the make-up of the consortium; however, combined species may not interact, either positively or negatively, meaning that conclusions may not be as translatable. In contrast, consortia that are allowed to emerge based on existing interactions between bacterial species, those whose constituent members are not chosen by the user, have a better chance of representing native interactions, or at least, interactions representative of the conditions that they were cultured under, but the complete taxonomic and genomic potential of the community is difficult to determine, and detailed experiments on individual members are not possible. Here, we take a combined approach where consortia are allowed to develop through several weeks of passaging followed by isolation of species and recombination. This maximized our chances of generating a consortium reflecting true interactions while also giving us a great deal of control over community makeup, experimental design and data related to the role of each species.

We noted if there was a pH change or the production of white precipitate (later confirmed to be CaCO_3_) every week and found that, especially in early weeks, there was a great deal of variability from week to week when looking at pH changes or precipitate formation. This is despite the fact that this is a closed system, and the introduction of new species is not possible. Since we re-passaged the microbes each week, we were essentially allowing a new consortium to assemble with each transfer. Several studies have examined the role of stochasticity in consortium or community assembly. One recent review laid out four elements of community assembly [52]; of these selection (ecological factors that alter community structure due to differences in fitness) and drift (species identity with regard to the relative abundance of all species in the community) apply most strongly to the development of CSC-A. According to the authors, selection is inherently not stochastic while drift inherently is stochastic. Stochastic effects of community drift may be a main driver as to why carbonate production and pH changes varied from week to week even in the same medium and community replicate. Slightly different changes in drift and, possibly, selection, led to slightly different consortia, the downstream effect of which was major changes in community phenotypes (CaCO_3_ production). These experiments emphasize the need to cast a wide net when attempting to develop communities with certain characteristics and allow time for communities to stabilize. Both time and diversity of starting conditions are needed to counteract the inherent stochasticity of community development and increase the chances of finding a community that fits the needs of the experiment. Other studies have also found that stochasticity is a main driver in community assembly and even that pH changes, a major factor in these experiments specifically, can significantly affect the stochastic assembly of communities [53, 54].

Despite the stochasticity we see in the consortia phenotypes across the range of passaging, the final consortia had many of the same species. This suggests that the difference in phenotypes that we see is not just a reflection of taxonomic differences in the consortia but also a function of which genes and processes are being expressed at the consortium level. Such phenotypic outcomes are the result of a complex set of inputs, and other studies have also found that microbial communities of similar taxonomies can have widely different sets of expressed genes and functions [55]. We also consistently saw the emergence of the five species used in our final CSC-A, despite differences between media types and replicates. This strongly suggests that these species have a suite of interactions that allows them to grow together (they emerge repeatedly from the native soil microbiome). This is another argument in favor of multiple evolutions with similar or identical conditions in such experiments. If the same set of species consistently emerge, despite the random chance that is a function of these enrichments, this is a strong argument that they should be examined further. We hypothesize that these interactions are one of the reasons why we see significantly higher amounts of CaCO_3_ in CSC-A versus any of the constituent species or even the combination of all species.

The use of microbial communities to sequester carbon in soil has been considered a carbon mitigation strategy for several years [56]. However, there are significant hurdles that stand between the development of a community in a laboratory setting and the application of this community, or the fundamental knowledge gained, to the field [57, 58]. Here, we are adding significant carbon to the medium in the form of B4 (containing tryptone and yeast extract) as well as the urea itself. In addition, nickel is being added as well, a well-known urease cofactor [38]. We see less CaCO_3_ under other kinds of medium (e.g., B4) which shows that available metal ions and nutrients have major effects on CaCO_3_ production. Such knowledge could help us target downstream application studies, focusing on systems with free calcium or nickel and higher inputs of urea (from fertilizer for example). The addition of carbon also means there is likely significant respiration from the community, depending on carbon use efficiency [59]. Stored carbon in the form of carbonate (or microbial biomass and necromass) must exceed the amount respired for long-term storage to be feasible. In addition, the added carbon in these experiments speaks to the possibility that there may not be enough nitrogen or carbon sources in native soil to stimulate robust growth and thus carbonate formation. Pairing species driving CaCO_3_ production with plants whose exudates could be a carbon and nitrogen source, especially those that have deep root systems [60], will be a critical link to capturing carbon directly from the atmosphere, putting it into soil as exudates or litter and storing it long-term via MICP deep in the soil. Sufficient minerals must also exist in the soil to lock the carbonate into CaCO_3_, or other forms that are inaccessible to metabolism, and the soil pH must be high enough (~ 7.5 to 8) for the CaCO_3_ to precipitate. Finally, the work we show here focuses on a very simple and artificial system compared to the complete soil microbiome. Our discoveries must first be confirmed in more complex and natural environments, linking our laboratory and field work, before we take the next steps to modify these systems to harness them for carbonate production.

Challenges remain in harnessing the soil microbiome to store carbon long-term, but a deeper understanding of the community-level processes involved is critical to addressing these challenges. The development and analysis of CSC-A will allow the scientific community to start to fill these knowledge gaps. We will be able to explore the role not only of individual species, but also of the interactions between them and how these effects change with shifts in carbon source, pH, and environment (liquid cultivation vs. back in the native soil). As we gather more knowledge on how species interact to sequester carbon, we will be better equipped to implement measures to enhance the formation of inorganic carbon in the soil. This could include using knowledge gained to drive the native soil microbiome to carry this out, or through the direct application of carbon capturing communities to soils, in a manner similar to what has already been seen over the past decades with microbial communities that have revolutionized agriculture and plant growth.

## Supplementary Information

Below is the link to the electronic supplementary material.

ESM 1 Supplementary Material 1 (XLSX 18.3 KB)

ESM 2 Supplementary Material 2 (XLSX 21.2 KB)

ESM 3 Supplementary Material 3 (PPTX 25.7 MB)

## Data Availability

16S rRNA sequencing data will be posted publicly on PNNL’s DataHub site and is also available upon request.

## References

[1] Getahun GT, Etana A, Munkholm LJ, Kirchmann H (2021) Liming with CaCO3 or CaO affects aggregate stability and dissolved reactive phosphorus in a heavy clay subsoil. Soil Tillage Res 214:105162

[2] Naeimi M et al (2023) Soil stabilization for dunes fixation using microbially induced calcium carbonate precipitation. Geoderma 429:116183

[3] Wang S et al (2023) Bio-remediation of heavy metal-contaminated soil by microbial-induced carbonate precipitation (MICP)—a critical review. Sustainability 15(9):7622

[4] Lal R (2004) Soil carbon sequestration impacts on global climate change and food security. Science 304(5677):1623–1627 15192216 10.1126/science.1097396

[5] Okyay TO et al (2016) CO2 sequestration by ureolytic microbial consortia through microbially-induced calcite precipitation. Sci Total Environ 572:671–680 27524723 10.1016/j.scitotenv.2016.06.199

[6] Korenblum E et al (2020) Rhizosphere microbiome mediates systemic root metabolite exudation by root-to-root signaling. Proc Natl Acad Sci U S A 117(7):3874–3883 32015118 10.1073/pnas.1912130117 PMC7035606

[7] Park I, Seo Y-S, Mannaa M (2023) Recruitment of the rhizo-microbiome army: assembly determinants and engineering of the rhizosphere microbiome as a key to unlocking plant potential. Front Microbiol 14:1163832 37213524 10.3389/fmicb.2023.1163832 PMC10196466

[8] Zhu T, Dittrich M (2016) Carbonate precipitation through microbial activities in natural environment, and their potential in biotechnology: a review. Front Bioeng Biotechnol 4:4 26835451 10.3389/fbioe.2016.00004 PMC4718973

[9] Phillips AJ et al (2013) Engineered applications of ureolytic biomineralization: a review. Biofouling 29(6):715–733 23802871 10.1080/08927014.2013.796550

[10] Sparks DL, Singh B, Siebecker MG (2022) Environmental soil chemistry. Elsevier

[11] Zamanian K, Zhou J, Kuzyakov Y (2021) Soil carbonates: the unaccounted, irrecoverable carbon source. Geoderma 384:114817

[12] Dhami NK, Reddy MS, Mukherjee A (2013) Biomineralization of calcium carbonates and their engineered applications: a review. Front Microbiol 4:314 24194735 10.3389/fmicb.2013.00314 PMC3810791

[13] Fu T, Saracho AC, Haigh SK (2023) Microbially induced carbonate precipitation (MICP) for soil strengthening: a comprehensive review. Biogeotechnics 1(1):100002

[14] Stocks-Fischer S, Galinat JK, Bang SS (1999) Microbiological precipitation of CaCO3. Soil Biol Biochem 31(11):1563–1571

[15] Liu P et al (2021) Bioremediation of metal-contaminated soils by microbially-induced carbonate precipitation and its effects on ecotoxicity and long-term stability. Biochem Eng J 166:107856

[16] Feng Su J et al (2019) Performance and microbial community of simultaneous removal of NO3−-N, Cd2+ and Ca2+ in MBBR. J Environ Manage 250:109548 31521921 10.1016/j.jenvman.2019.109548

[17] Fujita Y et al (2000) Subscribed content calcium carbonate precipitation by ureolytic subsurface bacteria. Geomicrobiol J 17(4):305–318

[18] Liu X et al (2021) Density-dependent microbial calcium carbonate precipitation by drinking water bacteria via amino acid metabolism and biosorption. Water Res 202:117444 34314923 10.1016/j.watres.2021.117444

[19] Zhu X et al (2016) The large-scale process of microbial carbonate precipitation for nickel remediation from an industrial soil. Environ Pollut 219:149–155 27814530 10.1016/j.envpol.2016.10.047

[20] Occhipinti R, Boron WF (2019) Role of carbonic anhydrases and inhibitors in acid–base physiology: insights from mathematical modeling. Int J Mol Sci 20(15):3841 31390837 10.3390/ijms20153841 PMC6695913

[21] Abdelsamad R et al (2022) Evidencing the role of carbonic anhydrase in the formation of carbonate minerals by bacterial strains isolated from extreme environments in Qatar. Heliyon. 10.1016/j.heliyon.2022.e11151 36311368 10.1016/j.heliyon.2022.e11151 PMC9614864

[22] Supuran CT, Capasso C (2017) An overview of the bacterial carbonic anhydrases. Metabolites 7(4):56 29137134 10.3390/metabo7040056 PMC5746736

[23] Giri A, Pant D (2019) CO2 management using carbonic anhydrase producing microbes from western Indian Himalaya. Bioresour Technol Rep 8:100320

[24] Gorter FA, Manhart M, Ackermann M (2020) Understanding the evolution of interspecies interactions in microbial communities. Philos Trans R Soc B 375(1798):20190256 10.1098/rstb.2019.0256 PMC7133538 32200743

[25] Saraiva JP et al (2021) Mining synergistic microbial interactions: a roadmap on how to integrate multi-omics data. Microorganisms 9(4):840 33920040 10.3390/microorganisms9040840 PMC8070991

[26] Lozano GL et al (2019) Introducing THOR, a model microbiome for genetic dissection of community behavior. MBio 10(2). 10.1128/mbio.02846-18 10.1128/mBio.02846-18 PMC6401489 30837345

[27] McClure R et al (2022) Interaction networks are driven by community-responsive phenotypes in a chitin-degrading consortium of soil microbes. MSystems 7(5):e00372–e00422 36154140 10.1128/msystems.00372-22 PMC9599572

[28] McClure R et al (2020) Development and analysis of a stable, reduced complexity model soil microbiome. Front Microbiol 11:1987 32983014 10.3389/fmicb.2020.01987 PMC7479069

[29] Connolly J et al (2013) Construction of two ureolytic model organisms for the study of microbially induced calcium carbonate precipitation. J Microbiol Methods 94(3):290–299 23835134 10.1016/j.mimet.2013.06.028

[30] Bolyen E et al (2019) Reproducible, interactive, scalable and extensible microbiome data science using QIIME 2. Nat Biotechnol 37(8):852–857 31341288 10.1038/s41587-019-0209-9 PMC7015180

[31] Callahan BJ et al (2016) DADA2: high-resolution sample inference from Illumina amplicon data. Nat Methods 13(7):581–583 27214047 10.1038/nmeth.3869 PMC4927377

[32] Davis NM et al (2018) Simple statistical identification and removal of contaminant sequences in marker-gene and metagenomics data. Microbiome 6:1–14 30558668 10.1186/s40168-018-0605-2 PMC6298009

[33] McMurdie PJ, Holmes S (2013) Phyloseq: an R package for reproducible interactive analysis and graphics of microbiome census data. PLoS ONE 8(4):e61217 23630581 10.1371/journal.pone.0061217 PMC3632530

[34] Oksanen J (2010) Vegan: community ecology package. https://vegan.r-forge.r-project.org/. Accessed 10 Aug 2025

[35] Love MI, Huber W, Anders S (2014) Moderated estimation of fold change and dispersion for RNA-seq data with DESeq2. Genome Biol 15(12):1–21 10.1186/s13059-014-0550-8 PMC4302049 25516281

[36] Team RC (2016) R: a language and environment for statistical computing. R Foundation for Statistical Computing, Vienna, Austria. https://www.R-project.org/. Accessed 11 Aug 2025

[37] Yin T, Cook D, Lawrence M (2012) Ggbio: an R package for extending the grammar of graphics for genomic data. Genome Biol 13(8):R77 22937822 10.1186/gb-2012-13-8-r77 PMC4053745

[38] Mazzei L, Musiani F, Ciurli S (2020) The structure-based reaction mechanism of urease, a nickel dependent enzyme: tale of a long debate. J Biol Inorg Chem 25(6):829–845 32809087 10.1007/s00775-020-01808-w PMC7433671

[39] Yoshida N, Higashimura E, Saeki Y (2010) Catalytic biomineralization of fluorescent calcite by the thermophilic bacterium Geobacillus thermoglucosidasius. Appl Environ Microbiol 76(21):7322–7327 20851984 10.1128/AEM.01767-10 PMC2976237

[40] Zambare NM et al (2020) Mineralogy of microbially induced calcium carbonate precipitates formed using single cell drop-based microfluidics. Sci Rep 10(1):17535 33067478 10.1038/s41598-020-73870-y PMC7568533

[41] Morris BE et al (2013) Microbial syntrophy: interaction for the common good. FEMS Microbiol Rev 37(3):384–406 23480449 10.1111/1574-6976.12019

[42] Zhu X et al (2020) Metabolic dependencies govern microbial syntrophies during methanogenesis in an anaerobic digestion ecosystem. Microbiome 8(1):1–14 32061251 10.1186/s40168-019-0780-9 PMC7024554

[43] Letek M et al (2010) The genome of a pathogenic Rhodococcus: cooptive virulence underpinned by key gene acquisitions. PLoS Genet 6(9):e1001145 20941392 10.1371/journal.pgen.1001145 PMC2947987

[44] Xu J-L et al (2007) Rhodococcus qingshengii sp. nov., a carbendazim-degrading bacterium. Int J Syst Evol Microbiol 57(12):2754–2757 18048720 10.1099/ijs.0.65095-0

[45] Heyse J et al (2019) Coculturing bacteria leads to reduced phenotypic heterogeneities. Appl Environ Microbiol 85(8):e02814–e02818 30796063 10.1128/AEM.02814-18 PMC6450022

[46] Jean-Pierre F et al (2023) Community composition shapes microbial-specific phenotypes in a cystic fibrosis polymicrobial model system. Elife 12:e81604 36661299 10.7554/eLife.81604 PMC9897730

[47] McClure R et al (2023) Removal of primary nutrient degraders reduces growth of soil microbial communities with genomic redundancy. Front Microbiol 13:1046661 36762098 10.3389/fmicb.2022.1046661 PMC9902710

[48] Zuñiga C et al (2019) Environmental stimuli drive a transition from cooperation to competition in synthetic phototrophic communities. Nat Microbiol 4(12):2184–2191 31591554 10.1038/s41564-019-0567-6

[49] Herrera Paredes S et al (2018) Design of synthetic bacterial communities for predictable plant phenotypes. PLoS Biol 16(2):e2003962 29462153 10.1371/journal.pbio.2003962 PMC5819758

[50] Shayanthan A, Ordoñez PAC, Oresnik IJ (2022) The role of synthetic microbial communities (SynCom) in sustainable agriculture. Front Agron 4:58

[51] Zegeye EK et al (2019) Selection, succession, and stabilization of soil microbial consortia. mSystems. 10.1128/mSystems.00055-19 31098394 10.1128/mSystems.00055-19 PMC6517688

[52] Zhou J, Ning D (2017) Stochastic community assembly: does it matter in microbial ecology? Microbiol Mol Biol Rev 81(4). 10.1128/mmbr.00002-17 10.1128/MMBR.00002-17 PMC5706748 29021219

[53] Tripathi BM et al (2018) Soil pH mediates the balance between stochastic and deterministic assembly of bacteria. ISME J 12(4):1072–1083 29515169 10.1038/s41396-018-0082-4 PMC5864241

[54] Liu N et al (2021) Relative importance of deterministic and stochastic processes on soil microbial community assembly in temperate grasslands. Microorganisms 9(9):1929 34576824 10.3390/microorganisms9091929 PMC8469474

[55] Naylor D et al (2020) Deconstructing the soil microbiome into reduced-complexity functional modules. MBio 11(4):e01349–e01420 32636252 10.1128/mBio.01349-20 PMC7343995

[56] Wang Y et al (2023) Applications of microbial-induced carbonate precipitation: a state-of-the-art review. Biogeotechnics. 10.1016/j.bgtech.2023.100008

[57] Mason A et al (2023) Microbial solutions to soil carbon sequestration. J Clean Prod: 137993. 10.1016/j.jclepro.2023.137993

[58] Fierer N, Walsh CM (2023) Can we manipulate the soil microbiome to promote carbon sequestration in croplands? PLoS Biol 21(7):e3002207 37437031 10.1371/journal.pbio.3002207 PMC10337918

[59] Adingo S et al (2021) Variation of soil microbial carbon use efficiency (CUE) and its influence mechanism in the context of global environmental change: a review. PeerJ 9:e12131 34721956 10.7717/peerj.12131 PMC8522642

[60] Lamb A et al (2022) Bioenergy sorghum’s deep roots: a key to sustainable biomass production on annual cropland. GCB Bioenergy 14(2):132–156

