# Supplementary material for: Community Dynamics Drive Calcium Carbonate Production in an Enriched Consortium of Soil Microbes
Source: Microb Ecol. 2025 Dec 23;89(1):32. doi: 10.1007/s00248-025-02632-y (PMC12808153; doi:10.1007/s00248-025-02632-y)
Supplement: Supplementary file 3 — Supplementary Material 3 (PPTX 25.7 MB) [file 248_2025_2632_MOESM3_ESM.pptx]

## Slide 1
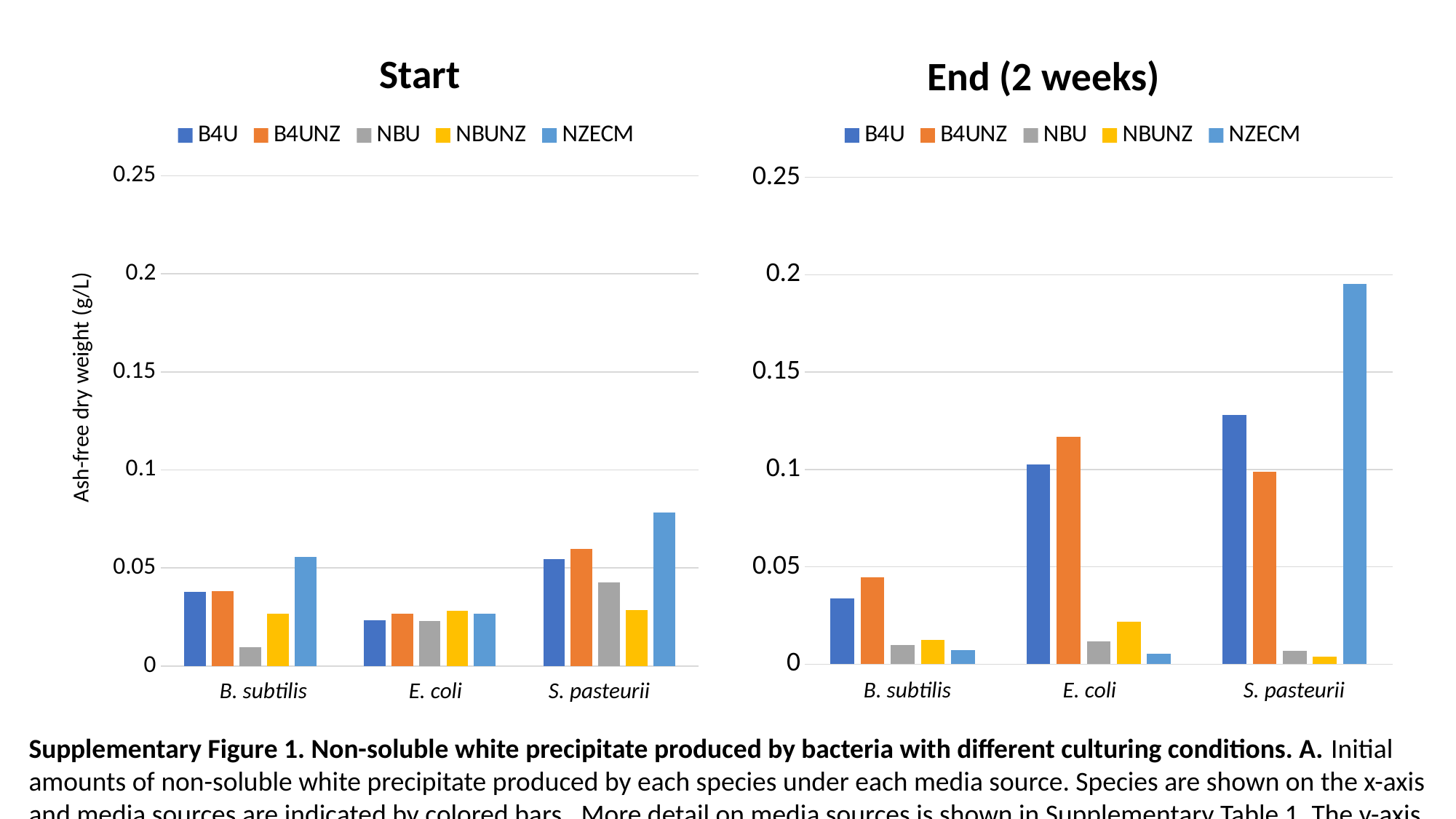

Start
End (2 weeks)
### Chart
| Category | | | | | |
|---|---|---|---|---|---|
| B. subtilis168_B4U | 0.03774999999999995 | 0.03796999999999984 | 0.00955000000000017 | 0.026570000000000205 | 0.05554999999999999 |
| E. coli MJK2_B4U | 0.023379999999999956 | 0.02648000000000006 | 0.022750000000000048 | 0.02808999999999995 | 0.026459999999999928 |
| S. pasteurii_B4U | 0.05432999999999999 | 0.059840000000000115 | 0.042649999999999855 | 0.028469999999999995 | 0.07811999999999997 |
### Chart
| Category | | | | | |
|---|---|---|---|---|---|
| B. subtilis168_B4U | 0.03383999999999987 | 0.04472000000000009 | 0.00969999999999982 | 0.012509999999999799 | 0.007299999999999862 |
| E. coli MJK2_B4U | 0.1024799999999999 | 0.11687000000000025 | 0.011740000000000084 | 0.021970000000000045 | 0.005210000000000048 |
| S. pasteurii_B4U | 0.12817000000000012 | 0.09889999999999999 | 0.007020000000000026 | 0.003730000000000011 | 0.19545999999999997 |Ash-free dry weight (g/L)
B. subtilis E. coli S. pasteurii
B. subtilis E. coli S. pasteurii
Supplementary Figure 1. Non-soluble white precipitate produced by bacteria with different culturing conditions. A. Initial amounts of non-soluble white precipitate produced by each species under each media source. Species are shown on the x-axis and media sources are indicated by colored bars. More detail on media sources is shown in Supplementary Table 1. The y-axis indicates ash-free dry weight in g/L. B. Similar to A but after a two-week incubation.

## Slide 2
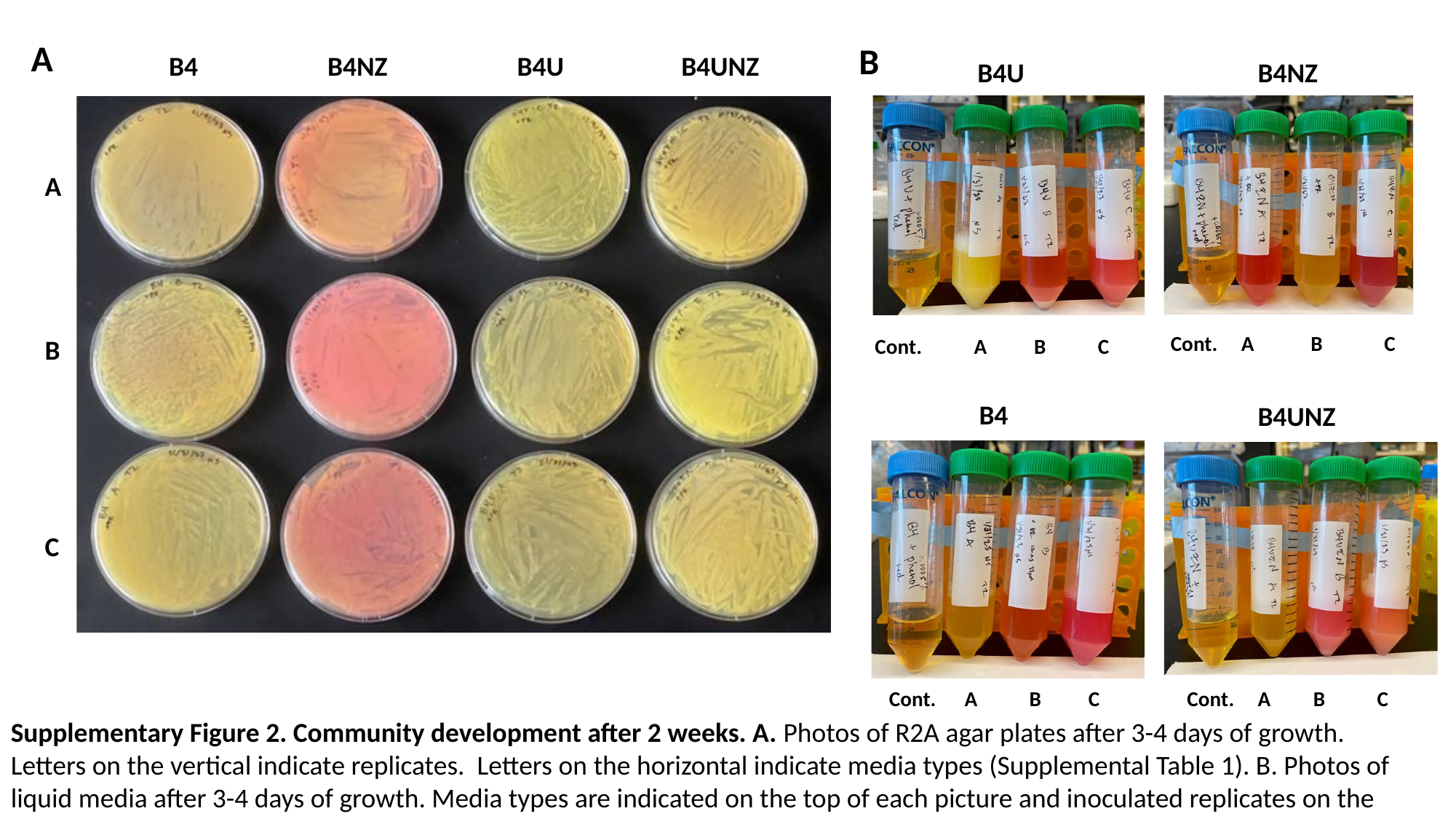

A
B
B4 B4NZ B4U B4UNZ
B4U
B4NZ
A
B
C
Cont. A B C
Cont. A B C
B4
B4UNZ
Cont. A B C
Cont. A B C
Supplementary Figure 2. Community development after 2 weeks. A. Photos of R2A agar plates after 3-4 days of growth. Letters on the vertical indicate replicates. Letters on the horizontal indicate media types (Supplemental Table 1). B. Photos of liquid media after 3-4 days of growth. Media types are indicated on the top of each picture and inoculated replicates on the bottom. Pink color indicates an increase in pH. “Cont.” indicates the uninoculated control.

## Slide 3
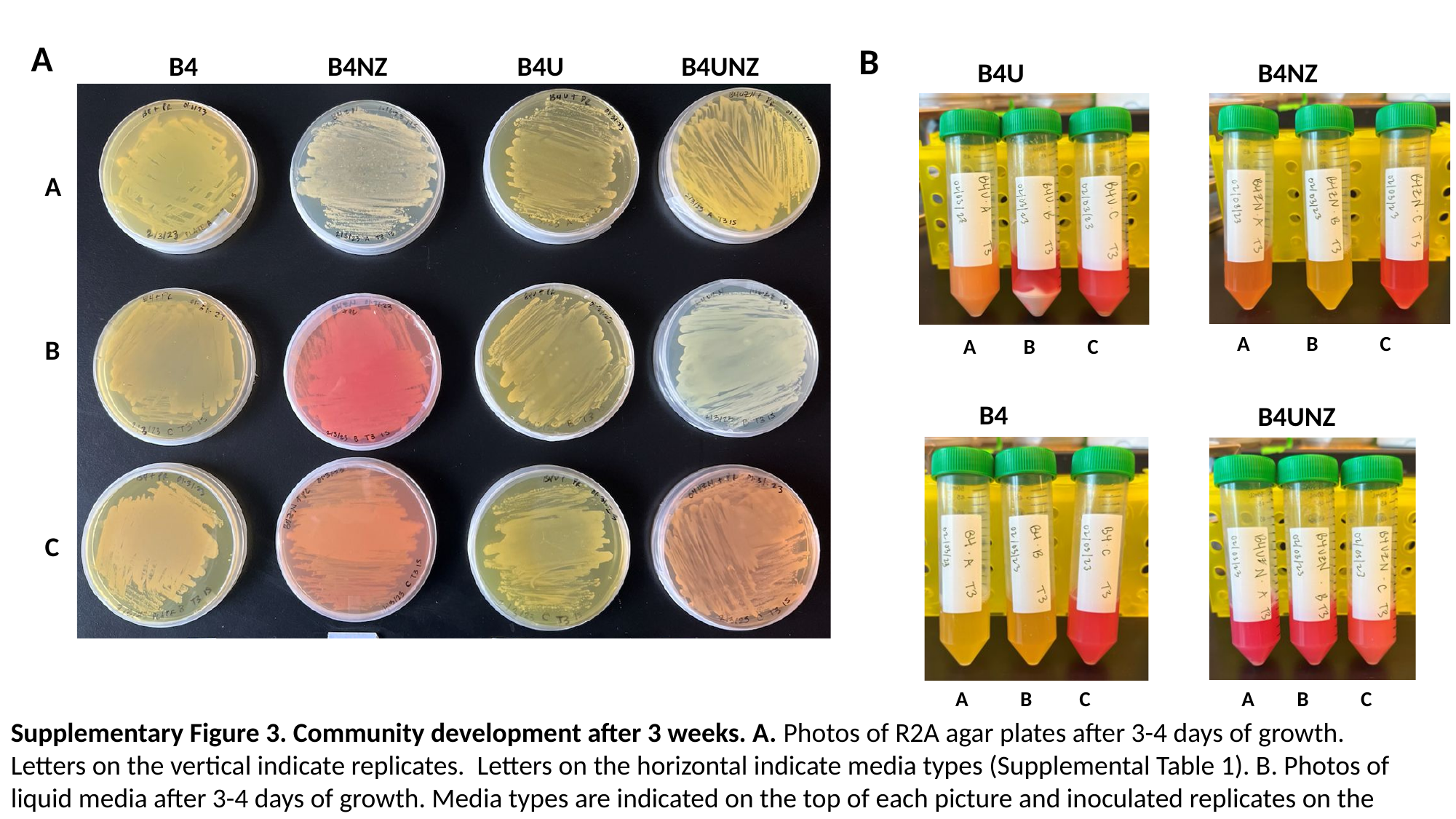

A
B
B4 B4NZ B4U B4UNZ
B4U
B4NZ
A
B
C
 A B C
 A B C
B4
B4UNZ
 A B C
 A B C
Supplementary Figure 3. Community development after 3 weeks. A. Photos of R2A agar plates after 3-4 days of growth. Letters on the vertical indicate replicates. Letters on the horizontal indicate media types (Supplemental Table 1). B. Photos of liquid media after 3-4 days of growth. Media types are indicated on the top of each picture and inoculated replicates on the bottom. Pink color indicates an increase in pH. “Cont.” indicates the uninoculated control.

## Slide 4
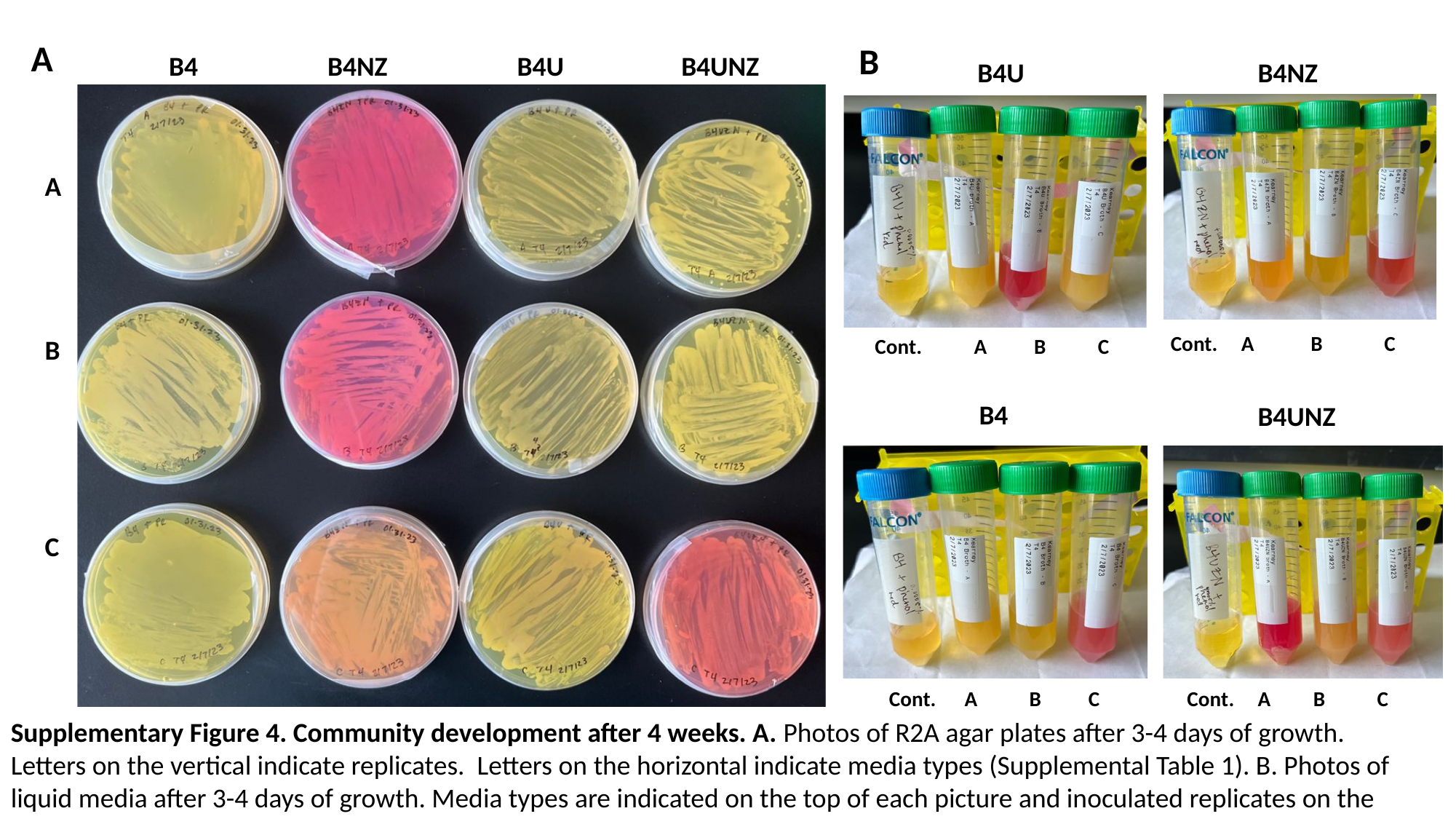

A
B
B4 B4NZ B4U B4UNZ
B4U
B4NZ
A
B
C
Cont. A B C
Cont. A B C
B4
B4UNZ
Cont. A B C
Cont. A B C
Supplementary Figure 4. Community development after 4 weeks. A. Photos of R2A agar plates after 3-4 days of growth. Letters on the vertical indicate replicates. Letters on the horizontal indicate media types (Supplemental Table 1). B. Photos of liquid media after 3-4 days of growth. Media types are indicated on the top of each picture and inoculated replicates on the bottom. Pink color indicates an increase in pH. Due to an oversight the uninoculated control tube was not photographed on this passage.

## Slide 5
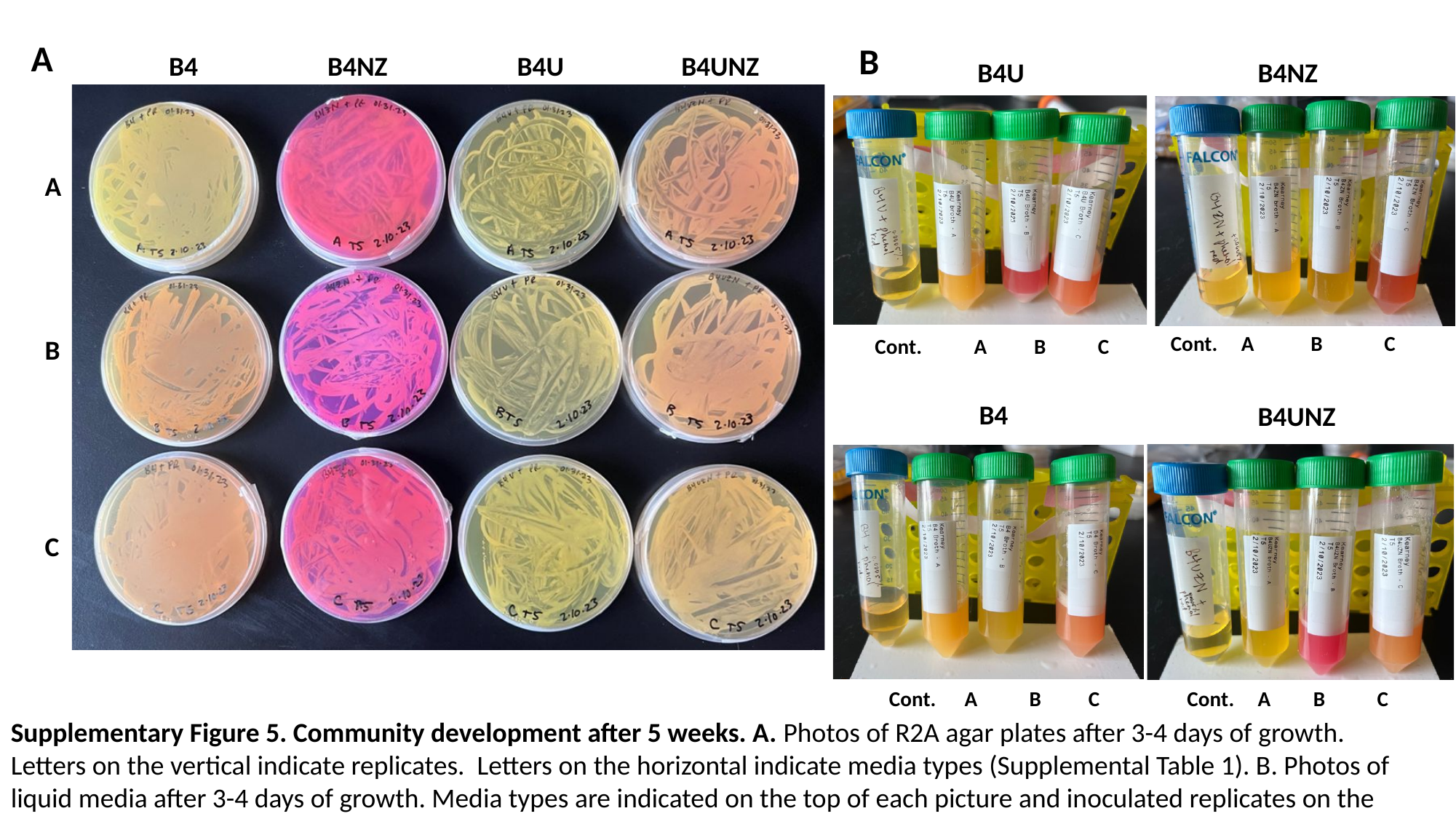

A
B
B4 B4NZ B4U B4UNZ
B4U
B4NZ
A
B
C
Cont. A B C
Cont. A B C
B4
B4UNZ
Cont. A B C
Cont. A B C
Supplementary Figure 5. Community development after 5 weeks. A. Photos of R2A agar plates after 3-4 days of growth. Letters on the vertical indicate replicates. Letters on the horizontal indicate media types (Supplemental Table 1). B. Photos of liquid media after 3-4 days of growth. Media types are indicated on the top of each picture and inoculated replicates on the bottom. Pink color indicates an increase in pH. “Cont.” indicates the uninoculated control.

## Slide 6
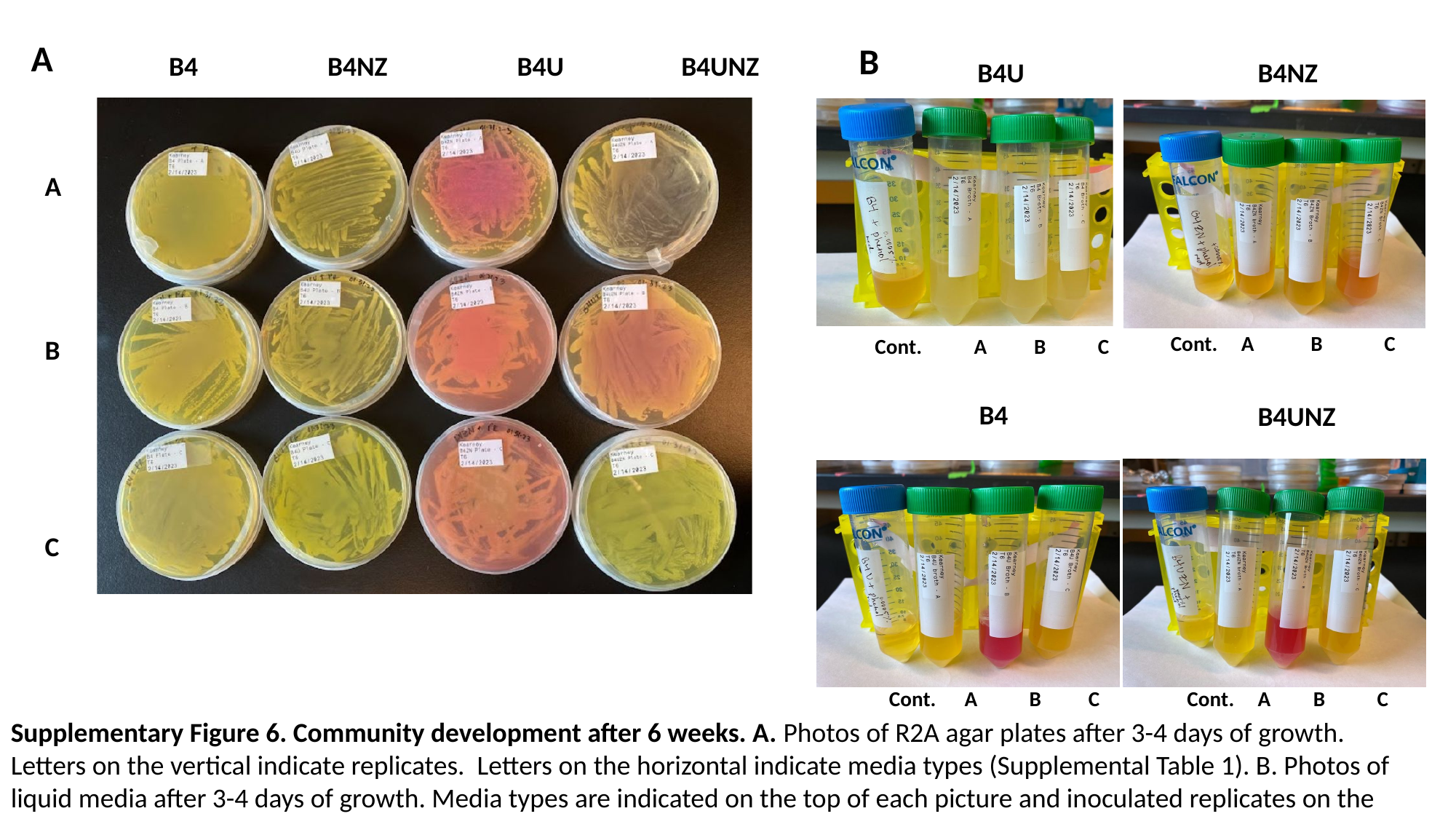

A
B
B4 B4NZ B4U B4UNZ
B4U
B4NZ
A
B
C
Cont. A B C
Cont. A B C
B4
B4UNZ
Cont. A B C
Cont. A B C
Supplementary Figure 6. Community development after 6 weeks. A. Photos of R2A agar plates after 3-4 days of growth. Letters on the vertical indicate replicates. Letters on the horizontal indicate media types (Supplemental Table 1). B. Photos of liquid media after 3-4 days of growth. Media types are indicated on the top of each picture and inoculated replicates on the bottom. Pink color indicates an increase in pH. “Cont.” indicates the uninoculated control.

## Slide 7
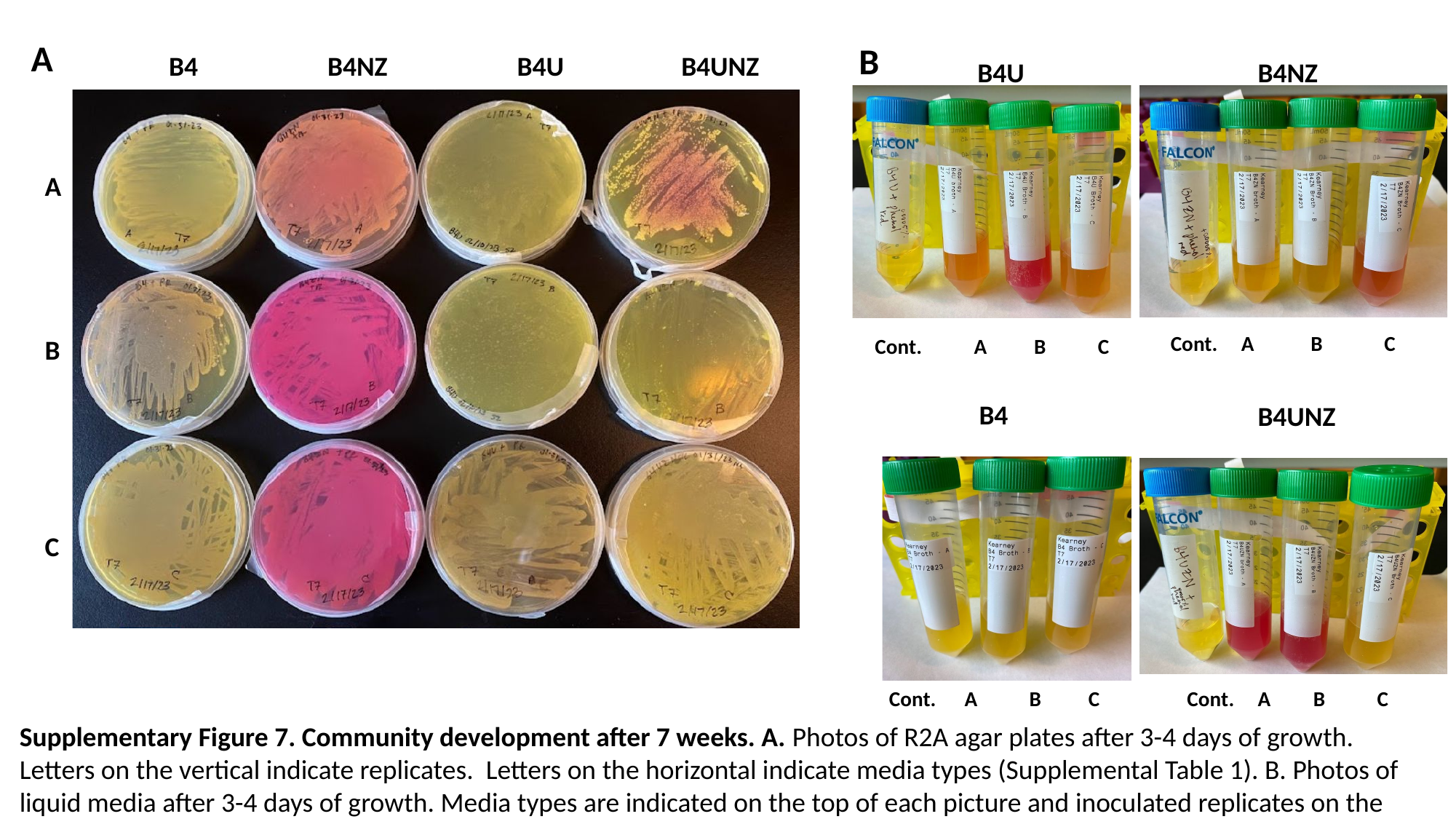

A
B
B4 B4NZ B4U B4UNZ
B4U
B4NZ
A
B
C
Cont. A B C
Cont. A B C
B4
B4UNZ
Cont. A B C
Cont. A B C
Supplementary Figure 7. Community development after 7 weeks. A. Photos of R2A agar plates after 3-4 days of growth. Letters on the vertical indicate replicates. Letters on the horizontal indicate media types (Supplemental Table 1). B. Photos of liquid media after 3-4 days of growth. Media types are indicated on the top of each picture and inoculated replicates on the bottom. Pink color indicates an increase in pH. “Cont.” indicates the uninoculated control.

## Slide 8
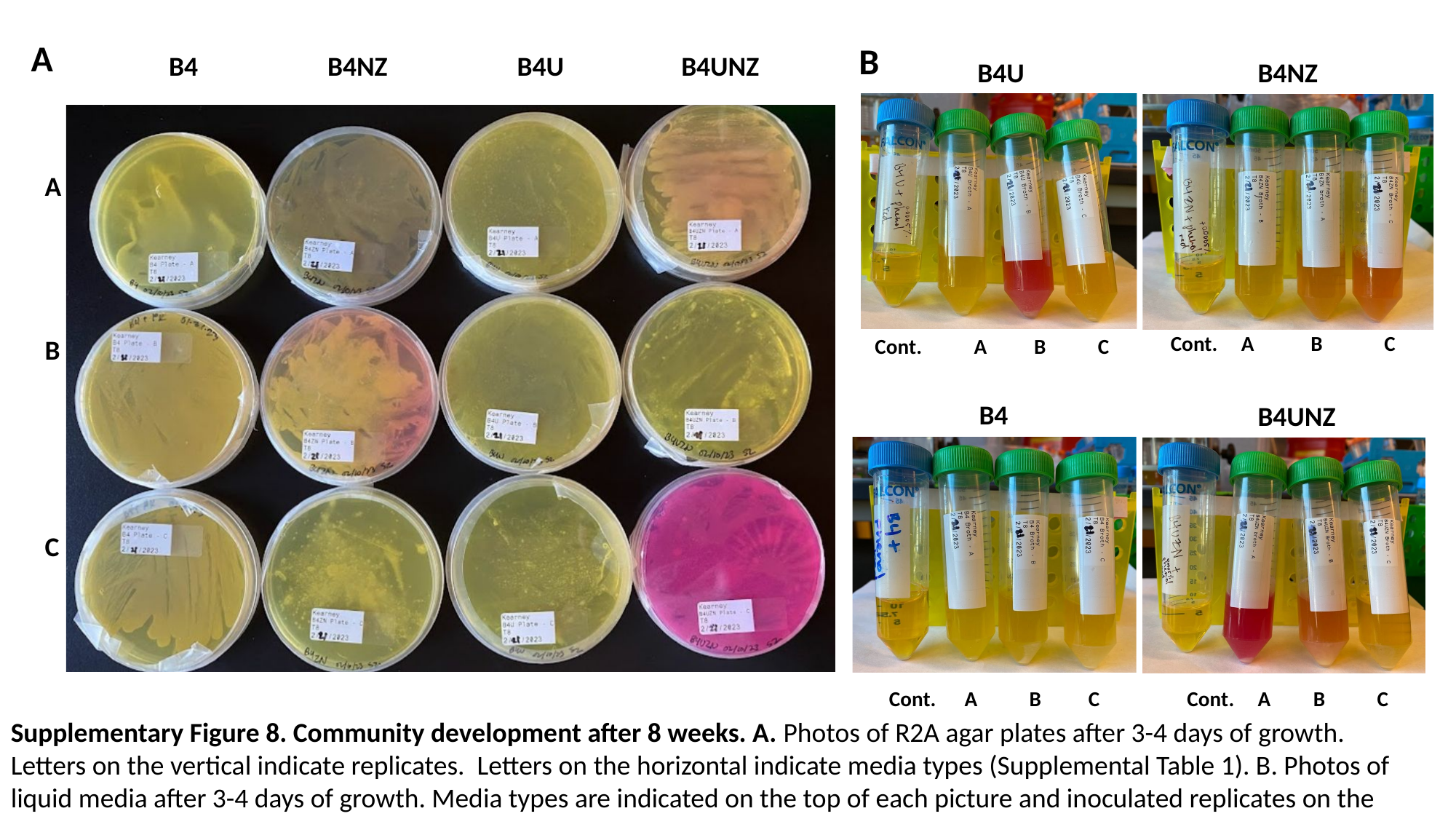

A
B
B4 B4NZ B4U B4UNZ
B4U
B4NZ
A
B
C
Cont. A B C
Cont. A B C
B4
B4UNZ
Cont. A B C
Cont. A B C
Supplementary Figure 8. Community development after 8 weeks. A. Photos of R2A agar plates after 3-4 days of growth. Letters on the vertical indicate replicates. Letters on the horizontal indicate media types (Supplemental Table 1). B. Photos of liquid media after 3-4 days of growth. Media types are indicated on the top of each picture and inoculated replicates on the bottom. Pink color indicates an increase in pH. “Cont.” indicates the uninoculated control.

## Slide 9
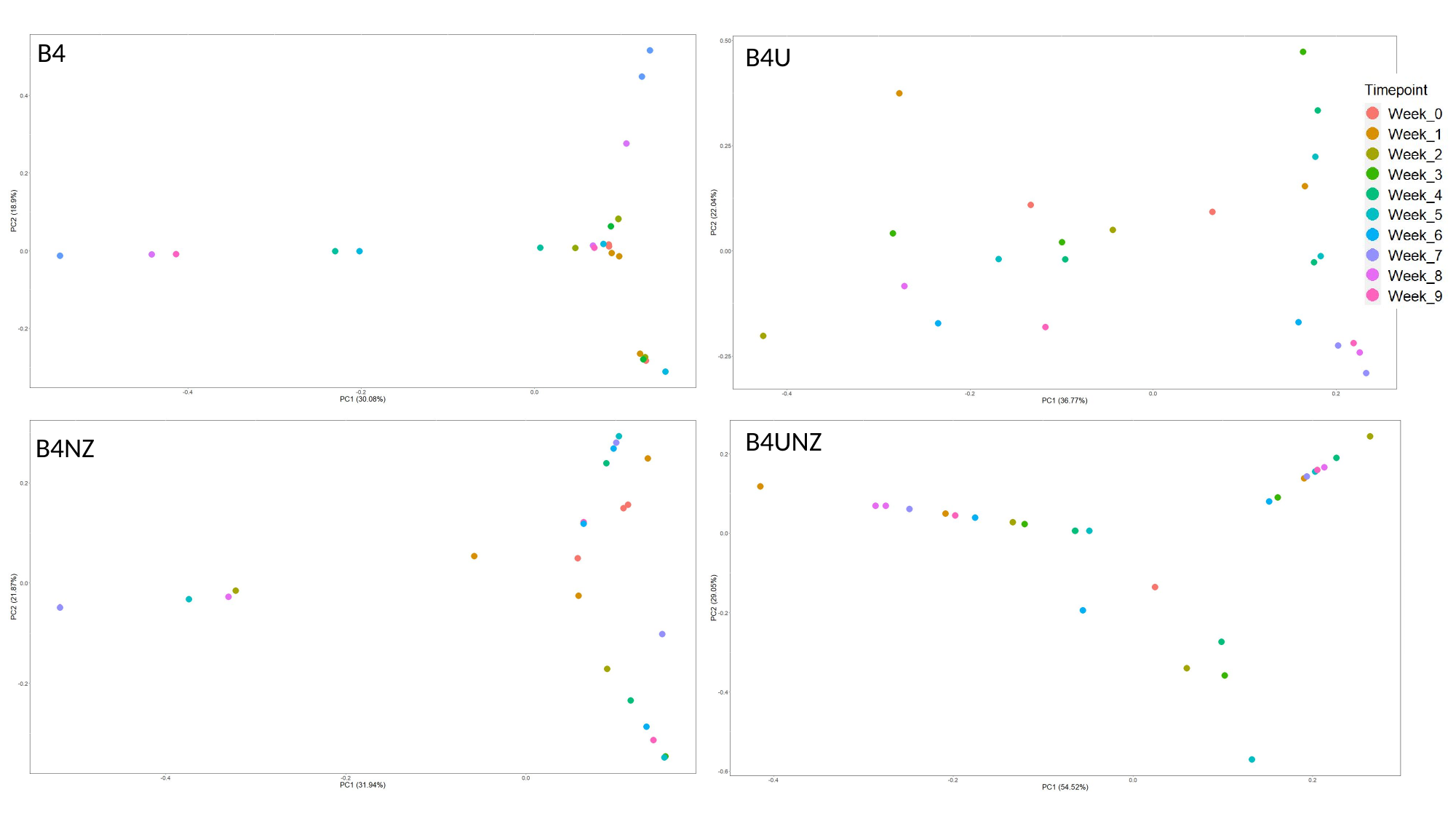

B4
B4U
B4UNZ
B4NZ
Supplementary Figure 9. PCA analysis of community development over time in liquid media. Four PCA plots are shown, one for each media type tested (labels in upper left). Markers of the same color indicate replicate communities with the color or the markers indicated passage number.

## Slide 10
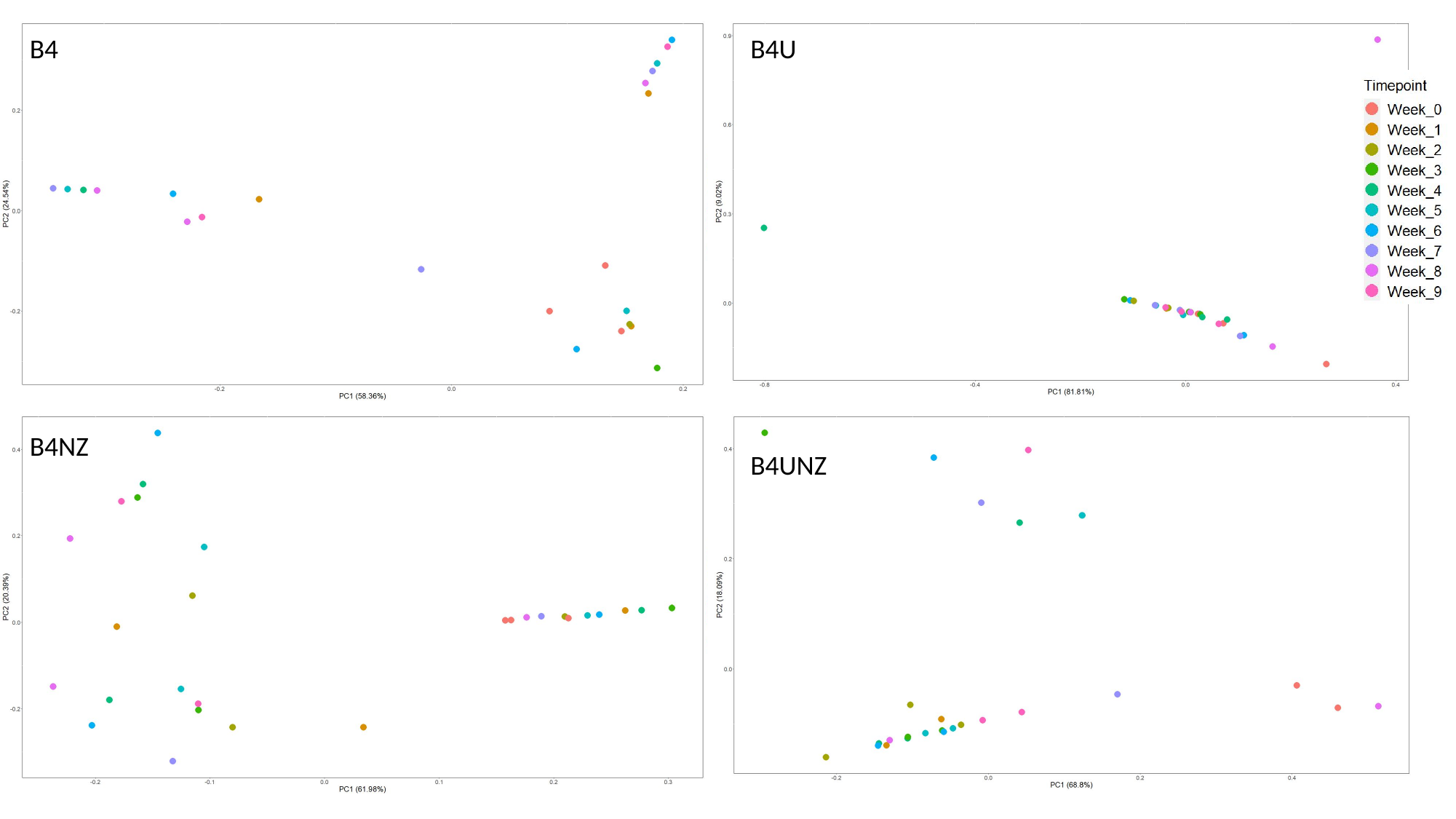

B4
B4U
B4NZ
B4UNZ
Supplementary Figure 10. PCA analysis of community development over time in agar plate media. Four PCA plots are shown, one for each media type tested (labels in upper left). Markers of the same color indicate replicate communities with the color or the markers indicated passage number.

## Slide 11
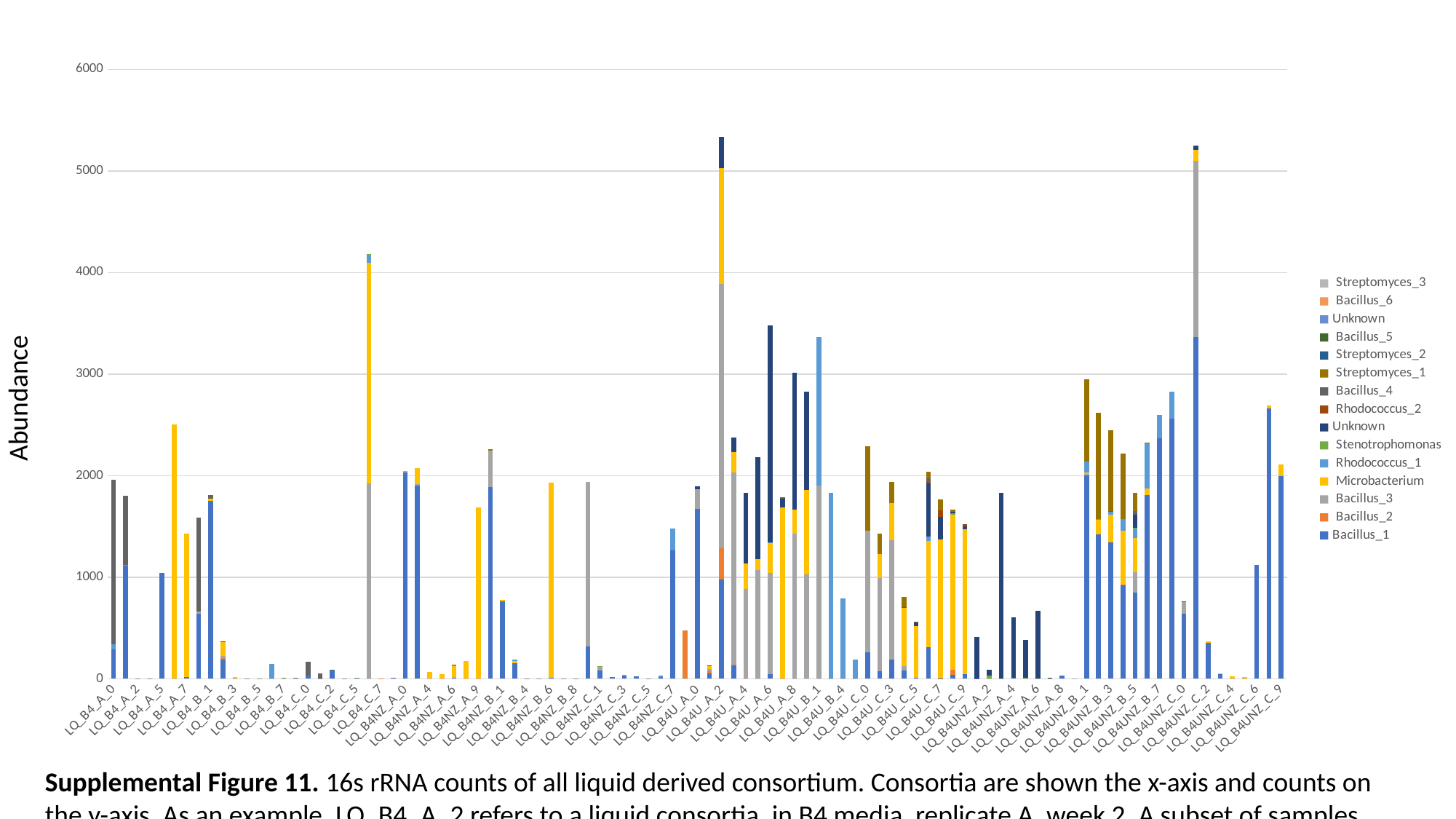

### Chart
| Category | Bacillus_1 | Bacillus_2 | Bacillus_3 | Microbacterium | Rhodococcus_1 | Stenotrophomonas | Unknown | Rhodococcus_2 | Bacillus_4 | Streptomyces_1 | Streptomyces_2 | Bacillus_5 | Unknown | Bacillus_6 | Streptomyces_3 |
|---|---|---|---|---|---|---|---|---|---|---|---|---|---|---|---|
| LQ_B4_A_0 | 288.0 | 0.0 | 0.0 | 0.0 | 54.0 | 0.0 | 0.0 | 0.0 | 1614.0 | 0.0 | 0.0 | 0.0 | 0.0 | 0.0 | 0.0 |
| LQ_B4_A_1 | 1110.0 | 0.0 | 0.0 | 0.0 | 9.0 | 0.0 | 0.0 | 0.0 | 685.0 | 0.0 | 0.0 | 0.0 | 0.0 | 0.0 | 0.0 |
| LQ_B4_A_2 | 0.0 | 0.0 | 0.0 | 0.0 | 0.0 | 0.0 | 0.0 | 0.0 | 0.0 | 0.0 | 0.0 | 0.0 | 0.0 | 0.0 | 0.0 |
| LQ_B4_A_4 | 0.0 | 0.0 | 0.0 | 0.0 | 0.0 | 0.0 | 0.0 | 0.0 | 0.0 | 0.0 | 0.0 | 0.0 | 0.0 | 0.0 | 0.0 |
| LQ_B4_A_5 | 1045.0 | 0.0 | 0.0 | 0.0 | 0.0 | 0.0 | 0.0 | 0.0 | 0.0 | 0.0 | 0.0 | 0.0 | 0.0 | 0.0 | 0.0 |
| LQ_B4_A_6 | 0.0 | 0.0 | 0.0 | 2505.0 | 0.0 | 0.0 | 0.0 | 0.0 | 0.0 | 0.0 | 0.0 | 0.0 | 0.0 | 0.0 | 0.0 |
| LQ_B4_A_7 | 19.0 | 0.0 | 0.0 | 1412.0 | 0.0 | 0.0 | 0.0 | 0.0 | 0.0 | 0.0 | 0.0 | 0.0 | 0.0 | 0.0 | 0.0 |
| LQ_B4_B_0 | 640.0 | 0.0 | 21.0 | 0.0 | 0.0 | 0.0 | 0.0 | 0.0 | 923.0 | 0.0 | 0.0 | 0.0 | 0.0 | 0.0 | 0.0 |
| LQ_B4_B_1 | 1749.0 | 0.0 | 0.0 | 24.0 | 0.0 | 0.0 | 0.0 | 0.0 | 39.0 | 0.0 | 0.0 | 0.0 | 0.0 | 0.0 | 0.0 |
| LQ_B4_B_2 | 187.0 | 15.0 | 20.0 | 138.0 | 0.0 | 0.0 | 0.0 | 0.0 | 0.0 | 0.0 | 7.0 | 0.0 | 0.0 | 0.0 | 0.0 |
| LQ_B4_B_3 | 0.0 | 0.0 | 0.0 | 19.0 | 0.0 | 0.0 | 0.0 | 0.0 | 0.0 | 0.0 | 0.0 | 0.0 | 0.0 | 0.0 | 0.0 |
| LQ_B4_B_4 | 0.0 | 0.0 | 0.0 | 0.0 | 0.0 | 0.0 | 0.0 | 0.0 | 0.0 | 0.0 | 0.0 | 0.0 | 0.0 | 0.0 | 0.0 |
| LQ_B4_B_5 | 0.0 | 0.0 | 0.0 | 0.0 | 0.0 | 0.0 | 0.0 | 0.0 | 0.0 | 0.0 | 0.0 | 0.0 | 0.0 | 0.0 | 0.0 |
| LQ_B4_B_6 | 0.0 | 0.0 | 0.0 | 0.0 | 143.0 | 0.0 | 0.0 | 0.0 | 0.0 | 0.0 | 0.0 | 0.0 | 0.0 | 0.0 | 0.0 |
| LQ_B4_B_7 | 0.0 | 0.0 | 0.0 | 0.0 | 6.0 | 5.0 | 0.0 | 0.0 | 0.0 | 0.0 | 0.0 | 0.0 | 0.0 | 0.0 | 0.0 |
| LQ_B4_B_8 | 0.0 | 0.0 | 0.0 | 0.0 | 7.0 | 0.0 | 0.0 | 0.0 | 0.0 | 0.0 | 0.0 | 0.0 | 0.0 | 0.0 | 0.0 |
| LQ_B4_C_0 | 28.0 | 0.0 | 0.0 | 0.0 | 0.0 | 0.0 | 0.0 | 0.0 | 140.0 | 0.0 | 0.0 | 0.0 | 0.0 | 0.0 | 0.0 |
| LQ_B4_C_1 | 0.0 | 0.0 | 0.0 | 0.0 | 0.0 | 0.0 | 0.0 | 0.0 | 52.0 | 0.0 | 0.0 | 0.0 | 0.0 | 0.0 | 0.0 |
| LQ_B4_C_2 | 84.0 | 0.0 | 0.0 | 0.0 | 0.0 | 0.0 | 0.0 | 0.0 | 7.0 | 0.0 | 0.0 | 0.0 | 0.0 | 0.0 | 0.0 |
| LQ_B4_C_3 | 0.0 | 0.0 | 0.0 | 0.0 | 0.0 | 0.0 | 0.0 | 0.0 | 0.0 | 0.0 | 0.0 | 0.0 | 0.0 | 0.0 | 0.0 |
| LQ_B4_C_5 | 0.0 | 0.0 | 0.0 | 0.0 | 13.0 | 0.0 | 0.0 | 0.0 | 0.0 | 0.0 | 0.0 | 0.0 | 0.0 | 0.0 | 0.0 |
| LQ_B4_C_6 | 0.0 | 0.0 | 1922.0 | 2174.0 | 63.0 | 22.0 | 0.0 | 0.0 | 0.0 | 0.0 | 0.0 | 0.0 | 0.0 | 0.0 | 0.0 |
| LQ_B4_C_7 | 0.0 | 4.0 | 0.0 | 0.0 | 0.0 | 0.0 | 0.0 | 0.0 | 0.0 | 0.0 | 0.0 | 0.0 | 0.0 | 0.0 | 0.0 |
| LQ_B4_C_8 | 10.0 | 0.0 | 0.0 | 0.0 | 0.0 | 0.0 | 0.0 | 0.0 | 0.0 | 0.0 | 0.0 | 0.0 | 0.0 | 0.0 | 0.0 |
| LQ_B4NZ_A_0 | 2032.0 | 0.0 | 10.0 | 0.0 | 0.0 | 0.0 | 0.0 | 0.0 | 0.0 | 0.0 | 0.0 | 0.0 | 0.0 | 0.0 | 0.0 |
| LQ_B4NZ_A_1 | 1903.0 | 0.0 | 16.0 | 156.0 | 0.0 | 0.0 | 0.0 | 0.0 | 0.0 | 0.0 | 0.0 | 0.0 | 0.0 | 0.0 | 0.0 |
| LQ_B4NZ_A_4 | 0.0 | 0.0 | 0.0 | 65.0 | 0.0 | 0.0 | 0.0 | 0.0 | 0.0 | 0.0 | 0.0 | 0.0 | 0.0 | 0.0 | 0.0 |
| LQ_B4NZ_A_5 | 0.0 | 0.0 | 0.0 | 43.0 | 0.0 | 0.0 | 0.0 | 0.0 | 0.0 | 0.0 | 0.0 | 0.0 | 0.0 | 0.0 | 0.0 |
| LQ_B4NZ_A_6 | 12.0 | 0.0 | 0.0 | 116.0 | 0.0 | 7.0 | 0.0 | 0.0 | 0.0 | 4.0 | 0.0 | 0.0 | 0.0 | 0.0 | 0.0 |
| LQ_B4NZ_A_7 | 0.0 | 0.0 | 0.0 | 175.0 | 0.0 | 0.0 | 0.0 | 0.0 | 0.0 | 0.0 | 0.0 | 0.0 | 0.0 | 0.0 | 0.0 |
| LQ_B4NZ_A_9 | 0.0 | 0.0 | 0.0 | 1689.0 | 0.0 | 0.0 | 0.0 | 0.0 | 0.0 | 0.0 | 0.0 | 0.0 | 0.0 | 0.0 | 0.0 |
| LQ_B4NZ_B_0 | 1891.0 | 0.0 | 358.0 | 0.0 | 0.0 | 0.0 | 0.0 | 0.0 | 0.0 | 10.0 | 0.0 | 0.0 | 0.0 | 0.0 | 0.0 |
| LQ_B4NZ_B_1 | 765.0 | 0.0 | 0.0 | 10.0 | 0.0 | 0.0 | 0.0 | 0.0 | 0.0 | 0.0 | 0.0 | 0.0 | 0.0 | 0.0 | 0.0 |
| LQ_B4NZ_B_2 | 156.0 | 0.0 | 0.0 | 20.0 | 15.0 | 0.0 | 0.0 | 0.0 | 0.0 | 0.0 | 0.0 | 0.0 | 0.0 | 0.0 | 0.0 |
| LQ_B4NZ_B_4 | 0.0 | 0.0 | 0.0 | 0.0 | 0.0 | 0.0 | 0.0 | 0.0 | 0.0 | 0.0 | 0.0 | 0.0 | 0.0 | 0.0 | 0.0 |
| LQ_B4NZ_B_5 | 0.0 | 0.0 | 0.0 | 0.0 | 0.0 | 0.0 | 0.0 | 0.0 | 0.0 | 0.0 | 0.0 | 0.0 | 0.0 | 0.0 | 0.0 |
| LQ_B4NZ_B_6 | 11.0 | 0.0 | 0.0 | 1917.0 | 0.0 | 0.0 | 0.0 | 0.0 | 0.0 | 0.0 | 0.0 | 0.0 | 0.0 | 0.0 | 0.0 |
| LQ_B4NZ_B_7 | 0.0 | 0.0 | 0.0 | 0.0 | 0.0 | 0.0 | 0.0 | 0.0 | 0.0 | 0.0 | 0.0 | 0.0 | 0.0 | 0.0 | 0.0 |
| LQ_B4NZ_B_8 | 0.0 | 0.0 | 0.0 | 0.0 | 0.0 | 0.0 | 0.0 | 0.0 | 0.0 | 0.0 | 0.0 | 0.0 | 0.0 | 0.0 | 0.0 |
| LQ_B4NZ_C_0 | 319.0 | 0.0 | 1618.0 | 0.0 | 0.0 | 0.0 | 0.0 | 0.0 | 0.0 | 0.0 | 0.0 | 0.0 | 0.0 | 0.0 | 0.0 |
| LQ_B4NZ_C_1 | 83.0 | 0.0 | 38.0 | 0.0 | 0.0 | 3.0 | 0.0 | 0.0 | 0.0 | 0.0 | 0.0 | 0.0 | 0.0 | 0.0 | 0.0 |
| LQ_B4NZ_C_2 | 15.0 | 0.0 | 0.0 | 0.0 | 0.0 | 0.0 | 0.0 | 0.0 | 0.0 | 0.0 | 0.0 | 0.0 | 0.0 | 0.0 | 0.0 |
| LQ_B4NZ_C_3 | 28.0 | 0.0 | 10.0 | 0.0 | 0.0 | 0.0 | 0.0 | 0.0 | 0.0 | 0.0 | 0.0 | 0.0 | 0.0 | 0.0 | 0.0 |
| LQ_B4NZ_C_4 | 26.0 | 0.0 | 0.0 | 0.0 | 0.0 | 0.0 | 0.0 | 0.0 | 0.0 | 0.0 | 0.0 | 0.0 | 0.0 | 0.0 | 0.0 |
| LQ_B4NZ_C_5 | 0.0 | 0.0 | 0.0 | 0.0 | 0.0 | 0.0 | 0.0 | 0.0 | 0.0 | 0.0 | 0.0 | 0.0 | 0.0 | 0.0 | 0.0 |
| LQ_B4NZ_C_6 | 17.0 | 0.0 | 0.0 | 0.0 | 15.0 | 0.0 | 0.0 | 0.0 | 0.0 | 0.0 | 0.0 | 0.0 | 0.0 | 0.0 | 0.0 |
| LQ_B4NZ_C_7 | 1261.0 | 0.0 | 0.0 | 0.0 | 215.0 | 0.0 | 0.0 | 0.0 | 0.0 | 0.0 | 0.0 | 0.0 | 0.0 | 0.0 | 0.0 |
| LQ_B4NZ_C_9 | 0.0 | 476.0 | 0.0 | 0.0 | 0.0 | 0.0 | 0.0 | 0.0 | 0.0 | 0.0 | 0.0 | 0.0 | 0.0 | 0.0 | 0.0 |
| LQ_B4U_A_0 | 1674.0 | 0.0 | 193.0 | 0.0 | 0.0 | 0.0 | 31.0 | 0.0 | 0.0 | 0.0 | 0.0 | 0.0 | 0.0 | 0.0 | 0.0 |
| LQ_B4U_A_1 | 52.0 | 23.0 | 18.0 | 31.0 | 0.0 | 0.0 | 0.0 | 0.0 | 0.0 | 0.0 | 5.0 | 0.0 | 0.0 | 0.0 | 0.0 |
| LQ_B4U_A_2 | 976.0 | 311.0 | 2599.0 | 1143.0 | 0.0 | 0.0 | 307.0 | 0.0 | 0.0 | 0.0 | 0.0 | 0.0 | 0.0 | 0.0 | 0.0 |
| LQ_B4U_A_3 | 131.0 | 11.0 | 1891.0 | 202.0 | 0.0 | 0.0 | 139.0 | 0.0 | 0.0 | 0.0 | 0.0 | 0.0 | 0.0 | 0.0 | 0.0 |
| LQ_B4U_A_4 | 0.0 | 0.0 | 887.0 | 251.0 | 0.0 | 0.0 | 691.0 | 0.0 | 0.0 | 0.0 | 0.0 | 0.0 | 0.0 | 0.0 | 0.0 |
| LQ_B4U_A_5 | 0.0 | 0.0 | 1067.0 | 112.0 | 0.0 | 0.0 | 1001.0 | 0.0 | 0.0 | 0.0 | 0.0 | 0.0 | 0.0 | 0.0 | 0.0 |
| LQ_B4U_A_6 | 43.0 | 0.0 | 1000.0 | 291.0 | 9.0 | 0.0 | 2136.0 | 0.0 | 0.0 | 0.0 | 0.0 | 0.0 | 0.0 | 0.0 | 0.0 |
| LQ_B4U_A_7 | 0.0 | 0.0 | 0.0 | 1690.0 | 0.0 | 0.0 | 83.0 | 0.0 | 11.0 | 0.0 | 0.0 | 0.0 | 0.0 | 0.0 | 0.0 |
| LQ_B4U_A_8 | 0.0 | 0.0 | 1429.0 | 235.0 | 0.0 | 0.0 | 1352.0 | 0.0 | 0.0 | 0.0 | 0.0 | 0.0 | 0.0 | 0.0 | 0.0 |
| LQ_B4U_A_9 | 0.0 | 0.0 | 1025.0 | 832.0 | 0.0 | 0.0 | 967.0 | 0.0 | 0.0 | 0.0 | 0.0 | 0.0 | 0.0 | 0.0 | 0.0 |
| LQ_B4U_B_1 | 0.0 | 0.0 | 1905.0 | 0.0 | 1463.0 | 0.0 | 0.0 | 0.0 | 0.0 | 0.0 | 0.0 | 0.0 | 0.0 | 0.0 | 0.0 |
| LQ_B4U_B_3 | 0.0 | 0.0 | 0.0 | 0.0 | 1832.0 | 0.0 | 0.0 | 0.0 | 0.0 | 0.0 | 0.0 | 0.0 | 0.0 | 0.0 | 0.0 |
| LQ_B4U_B_4 | 0.0 | 0.0 | 0.0 | 0.0 | 792.0 | 0.0 | 0.0 | 0.0 | 0.0 | 0.0 | 0.0 | 0.0 | 0.0 | 0.0 | 0.0 |
| LQ_B4U_B_5 | 0.0 | 0.0 | 0.0 | 0.0 | 191.0 | 0.0 | 0.0 | 0.0 | 0.0 | 0.0 | 0.0 | 0.0 | 0.0 | 0.0 | 0.0 |
| LQ_B4U_C_0 | 259.0 | 0.0 | 1196.0 | 0.0 | 0.0 | 0.0 | 0.0 | 0.0 | 0.0 | 834.0 | 0.0 | 0.0 | 0.0 | 0.0 | 0.0 |
| LQ_B4U_C_2 | 72.0 | 0.0 | 918.0 | 238.0 | 0.0 | 0.0 | 0.0 | 0.0 | 0.0 | 198.0 | 0.0 | 0.0 | 0.0 | 0.0 | 0.0 |
| LQ_B4U_C_3 | 188.0 | 0.0 | 1178.0 | 361.0 | 0.0 | 0.0 | 0.0 | 0.0 | 0.0 | 211.0 | 0.0 | 0.0 | 0.0 | 0.0 | 0.0 |
| LQ_B4U_C_4 | 78.0 | 0.0 | 43.0 | 582.0 | 0.0 | 0.0 | 0.0 | 4.0 | 0.0 | 95.0 | 0.0 | 0.0 | 0.0 | 0.0 | 0.0 |
| LQ_B4U_C_5 | 10.0 | 0.0 | 0.0 | 510.0 | 0.0 | 0.0 | 34.0 | 0.0 | 0.0 | 9.0 | 0.0 | 0.0 | 0.0 | 0.0 | 0.0 |
| LQ_B4U_C_6 | 314.0 | 0.0 | 0.0 | 1043.0 | 43.0 | 0.0 | 525.0 | 13.0 | 33.0 | 69.0 | 0.0 | 0.0 | 0.0 | 0.0 | 0.0 |
| LQ_B4U_C_7 | 2.0 | 0.0 | 0.0 | 1367.0 | 0.0 | 0.0 | 221.0 | 65.0 | 0.0 | 111.0 | 0.0 | 0.0 | 0.0 | 0.0 | 0.0 |
| LQ_B4U_C_8 | 35.0 | 55.0 | 0.0 | 1527.0 | 0.0 | 12.0 | 14.0 | 0.0 | 0.0 | 21.0 | 0.0 | 0.0 | 0.0 | 0.0 | 0.0 |
| LQ_B4U_C_9 | 44.0 | 0.0 | 0.0 | 1425.0 | 0.0 | 0.0 | 31.0 | 20.0 | 0.0 | 0.0 | 0.0 | 0.0 | 0.0 | 0.0 | 0.0 |
| LQ_B4UNZ_A_1 | 4.0 | 0.0 | 0.0 | 0.0 | 0.0 | 0.0 | 408.0 | 0.0 | 0.0 | 0.0 | 0.0 | 0.0 | 0.0 | 0.0 | 0.0 |
| LQ_B4UNZ_A_2 | 0.0 | 0.0 | 0.0 | 0.0 | 0.0 | 28.0 | 59.0 | 0.0 | 0.0 | 0.0 | 0.0 | 0.0 | 0.0 | 0.0 | 0.0 |
| LQ_B4UNZ_A_3 | 0.0 | 0.0 | 0.0 | 0.0 | 0.0 | 0.0 | 1827.0 | 0.0 | 0.0 | 0.0 | 0.0 | 0.0 | 0.0 | 0.0 | 0.0 |
| LQ_B4UNZ_A_4 | 6.0 | 0.0 | 0.0 | 0.0 | 0.0 | 0.0 | 601.0 | 0.0 | 0.0 | 0.0 | 0.0 | 0.0 | 0.0 | 0.0 | 0.0 |
| LQ_B4UNZ_A_5 | 0.0 | 0.0 | 0.0 | 0.0 | 0.0 | 10.0 | 373.0 | 0.0 | 0.0 | 0.0 | 0.0 | 0.0 | 0.0 | 0.0 | 0.0 |
| LQ_B4UNZ_A_6 | 0.0 | 0.0 | 0.0 | 0.0 | 0.0 | 0.0 | 668.0 | 0.0 | 0.0 | 0.0 | 0.0 | 0.0 | 0.0 | 0.0 | 0.0 |
| LQ_B4UNZ_A_7 | 0.0 | 0.0 | 0.0 | 0.0 | 0.0 | 0.0 | 8.0 | 0.0 | 0.0 | 0.0 | 0.0 | 0.0 | 0.0 | 0.0 | 0.0 |
| LQ_B4UNZ_A_8 | 32.0 | 0.0 | 0.0 | 0.0 | 0.0 | 0.0 | 0.0 | 0.0 | 0.0 | 0.0 | 0.0 | 0.0 | 0.0 | 0.0 | 0.0 |
| LQ_B4UNZ_A_9 | 0.0 | 0.0 | 5.0 | 0.0 | 0.0 | 0.0 | 0.0 | 0.0 | 0.0 | 0.0 | 0.0 | 0.0 | 0.0 | 0.0 | 0.0 |
| LQ_B4UNZ_B_1 | 2000.0 | 0.0 | 14.0 | 17.0 | 104.0 | 0.0 | 0.0 | 0.0 | 0.0 | 816.0 | 0.0 | 0.0 | 0.0 | 0.0 | 0.0 |
| LQ_B4UNZ_B_2 | 1424.0 | 0.0 | 0.0 | 140.0 | 11.0 | 0.0 | 0.0 | 0.0 | 0.0 | 1042.0 | 0.0 | 0.0 | 0.0 | 0.0 | 0.0 |
| LQ_B4UNZ_B_3 | 1342.0 | 0.0 | 0.0 | 270.0 | 31.0 | 0.0 | 0.0 | 7.0 | 0.0 | 794.0 | 0.0 | 0.0 | 0.0 | 0.0 | 0.0 |
| LQ_B4UNZ_B_4 | 924.0 | 6.0 | 0.0 | 525.0 | 116.0 | 0.0 | 0.0 | 0.0 | 0.0 | 645.0 | 0.0 | 0.0 | 0.0 | 0.0 | 0.0 |
| LQ_B4UNZ_B_5 | 847.0 | 0.0 | 200.0 | 341.0 | 88.0 | 9.0 | 129.0 | 0.0 | 34.0 | 181.0 | 0.0 | 0.0 | 0.0 | 0.0 | 0.0 |
| LQ_B4UNZ_B_6 | 1811.0 | 0.0 | 0.0 | 62.0 | 445.0 | 0.0 | 0.0 | 0.0 | 0.0 | 6.0 | 0.0 | 0.0 | 0.0 | 0.0 | 0.0 |
| LQ_B4UNZ_B_7 | 2369.0 | 0.0 | 0.0 | 0.0 | 230.0 | 0.0 | 0.0 | 0.0 | 0.0 | 0.0 | 0.0 | 0.0 | 0.0 | 0.0 | 0.0 |
| LQ_B4UNZ_B_8 | 2563.0 | 0.0 | 0.0 | 0.0 | 262.0 | 0.0 | 0.0 | 0.0 | 0.0 | 0.0 | 0.0 | 0.0 | 0.0 | 0.0 | 0.0 |
| LQ_B4UNZ_C_0 | 640.0 | 0.0 | 112.0 | 0.0 | 0.0 | 0.0 | 0.0 | 9.0 | 0.0 | 0.0 | 0.0 | 0.0 | 0.0 | 0.0 | 0.0 |
| LQ_B4UNZ_C_1 | 3365.0 | 0.0 | 1737.0 | 107.0 | 0.0 | 0.0 | 42.0 | 0.0 | 0.0 | 0.0 | 0.0 | 0.0 | 0.0 | 0.0 | 0.0 |
| LQ_B4UNZ_C_2 | 356.0 | 0.0 | 0.0 | 11.0 | 0.0 | 0.0 | 0.0 | 0.0 | 0.0 | 0.0 | 0.0 | 0.0 | 0.0 | 0.0 | 0.0 |
| LQ_B4UNZ_C_3 | 47.0 | 0.0 | 0.0 | 8.0 | 0.0 | 0.0 | 0.0 | 0.0 | 0.0 | 0.0 | 0.0 | 0.0 | 0.0 | 0.0 | 0.0 |
| LQ_B4UNZ_C_4 | 0.0 | 0.0 | 0.0 | 26.0 | 0.0 | 0.0 | 0.0 | 0.0 | 0.0 | 0.0 | 0.0 | 0.0 | 0.0 | 0.0 | 0.0 |
| LQ_B4UNZ_C_5 | 0.0 | 0.0 | 0.0 | 19.0 | 0.0 | 0.0 | 0.0 | 0.0 | 0.0 | 0.0 | 0.0 | 0.0 | 0.0 | 0.0 | 0.0 |
| LQ_B4UNZ_C_6 | 1119.0 | 0.0 | 0.0 | 0.0 | 0.0 | 0.0 | 0.0 | 0.0 | 0.0 | 0.0 | 0.0 | 0.0 | 0.0 | 0.0 | 0.0 |
| LQ_B4UNZ_C_8 | 2665.0 | 0.0 | 0.0 | 24.0 | 0.0 | 0.0 | 0.0 | 0.0 | 0.0 | 0.0 | 0.0 | 0.0 | 0.0 | 0.0 | 0.0 |
| LQ_B4UNZ_C_9 | 1997.0 | 0.0 | 0.0 | 116.0 | 0.0 | 0.0 | 0.0 | 0.0 | 0.0 | 0.0 | 0.0 | 0.0 | 0.0 | 0.0 | 0.0 |Abundance
Supplemental Figure 11. 16s rRNA counts of all liquid derived consortium. Consortia are shown the x-axis and counts on the y-axis. As an example, LQ_B4_A_2 refers to a liquid consortia, in B4 media, replicate A, week 2. A subset of samples gave poor sequencing results and are not displayed. 16s rRNA sequencing was not carried out on liquid controls as there was no bacterial growth.

## Slide 12
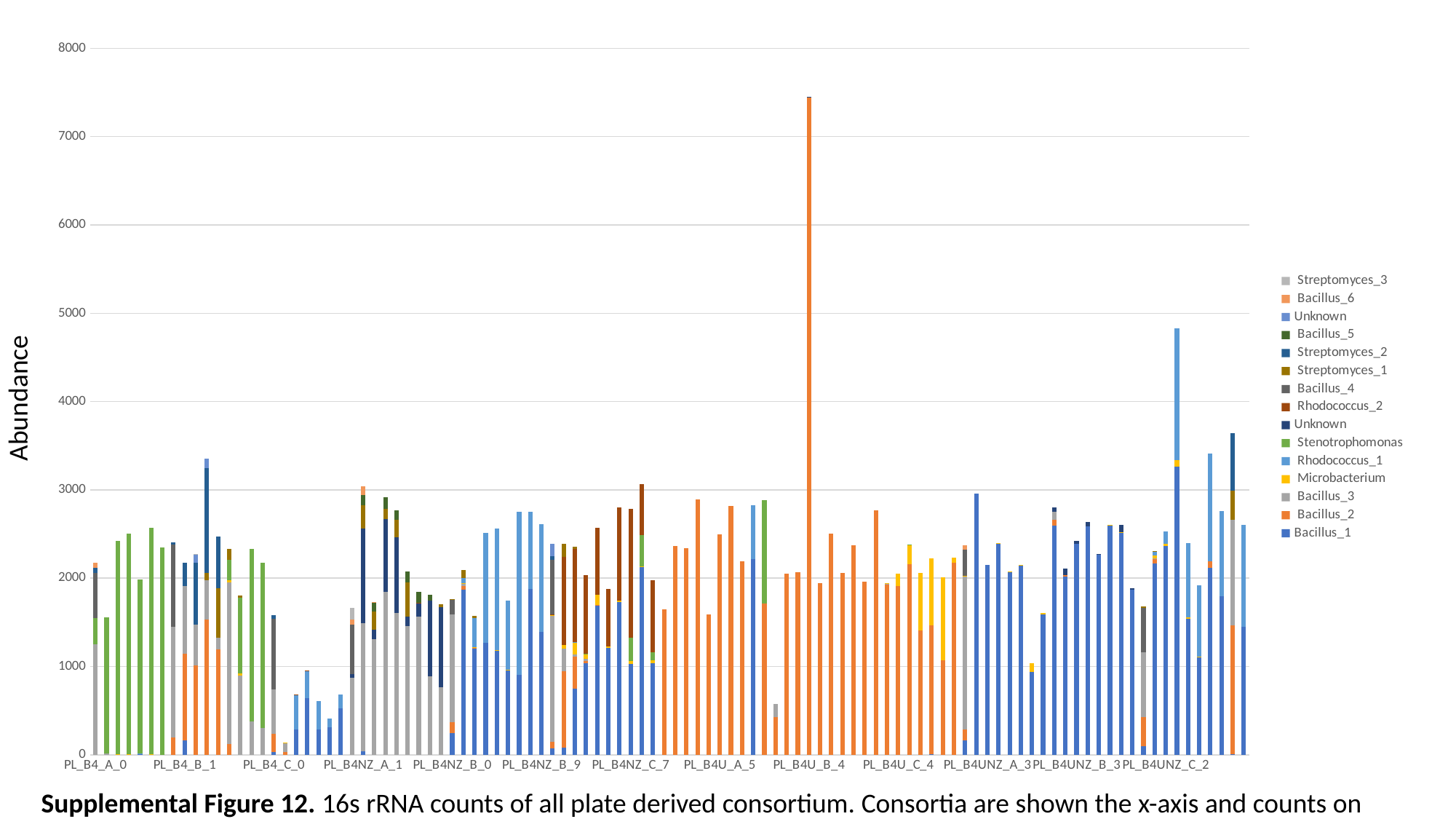

### Chart
| Category | Bacillus_1 | Bacillus_2 | Bacillus_3 | Microbacterium | Rhodococcus_1 | Stenotrophomonas | Unknown | Rhodococcus_2 | Bacillus_4 | Streptomyces_1 | Streptomyces_2 | Bacillus_5 | Unknown | Bacillus_6 | Streptomyces_3 |
|---|---|---|---|---|---|---|---|---|---|---|---|---|---|---|---|
| PL_B4_A_0 | 0.0 | 0.0 | 1248.0 | 0.0 | 0.0 | 303.0 | 0.0 | 0.0 | 512.0 | 0.0 | 53.0 | 0.0 | 0.0 | 58.0 | 0.0 |
| PL_B4_A_1 | 0.0 | 0.0 | 15.0 | 0.0 | 0.0 | 1540.0 | 0.0 | 0.0 | 0.0 | 0.0 | 0.0 | 0.0 | 0.0 | 0.0 | 0.0 |
| PL_B4_A_4 | 0.0 | 0.0 | 0.0 | 4.0 | 0.0 | 2416.0 | 0.0 | 0.0 | 0.0 | 0.0 | 0.0 | 0.0 | 0.0 | 0.0 | 0.0 |
| PL_B4_A_5 | 0.0 | 0.0 | 0.0 | 8.0 | 0.0 | 2497.0 | 0.0 | 0.0 | 0.0 | 0.0 | 0.0 | 0.0 | 0.0 | 0.0 | 0.0 |
| PL_B4_A_6 | 8.0 | 0.0 | 0.0 | 0.0 | 8.0 | 1970.0 | 0.0 | 0.0 | 0.0 | 0.0 | 0.0 | 0.0 | 0.0 | 0.0 | 0.0 |
| PL_B4_A_7 | 0.0 | 0.0 | 0.0 | 7.0 | 0.0 | 2565.0 | 0.0 | 0.0 | 0.0 | 0.0 | 0.0 | 0.0 | 0.0 | 0.0 | 0.0 |
| PL_B4_A_8 | 0.0 | 0.0 | 0.0 | 0.0 | 0.0 | 2345.0 | 0.0 | 0.0 | 0.0 | 0.0 | 0.0 | 0.0 | 0.0 | 0.0 | 0.0 |
| PL_B4_B_0 | 0.0 | 193.0 | 1255.0 | 0.0 | 0.0 | 0.0 | 0.0 | 0.0 | 930.0 | 0.0 | 24.0 | 0.0 | 0.0 | 0.0 | 0.0 |
| PL_B4_B_1 | 162.0 | 984.0 | 762.0 | 0.0 | 0.0 | 0.0 | 0.0 | 0.0 | 0.0 | 0.0 | 265.0 | 0.0 | 0.0 | 0.0 | 0.0 |
| PL_B4_B_2 | 0.0 | 1012.0 | 461.0 | 0.0 | 0.0 | 0.0 | 0.0 | 0.0 | 0.0 | 0.0 | 703.0 | 0.0 | 90.0 | 0.0 | 10.0 |
| PL_B4_B_3 | 0.0 | 1532.0 | 446.0 | 0.0 | 0.0 | 0.0 | 0.0 | 0.0 | 0.0 | 81.0 | 1188.0 | 0.0 | 108.0 | 0.0 | 0.0 |
| PL_B4_B_5 | 0.0 | 1195.0 | 127.0 | 0.0 | 0.0 | 0.0 | 0.0 | 0.0 | 0.0 | 563.0 | 588.0 | 0.0 | 0.0 | 0.0 | 0.0 |
| PL_B4_B_6 | 0.0 | 123.0 | 1826.0 | 24.0 | 0.0 | 238.0 | 0.0 | 0.0 | 0.0 | 122.0 | 0.0 | 0.0 | 0.0 | 0.0 | 0.0 |
| PL_B4_B_7 | 0.0 | 0.0 | 899.0 | 25.0 | 0.0 | 859.0 | 0.0 | 0.0 | 0.0 | 23.0 | 0.0 | 0.0 | 0.0 | 0.0 | 0.0 |
| PL_B4_B_8 | 0.0 | 0.0 | 380.0 | 0.0 | 0.0 | 1953.0 | 0.0 | 0.0 | 0.0 | 0.0 | 0.0 | 0.0 | 0.0 | 0.0 | 0.0 |
| PL_B4_B_9 | 0.0 | 0.0 | 303.0 | 0.0 | 0.0 | 1868.0 | 0.0 | 0.0 | 0.0 | 0.0 | 0.0 | 0.0 | 0.0 | 0.0 | 0.0 |
| PL_B4_C_0 | 31.0 | 206.0 | 505.0 | 0.0 | 0.0 | 0.0 | 0.0 | 0.0 | 794.0 | 0.0 | 42.0 | 0.0 | 0.0 | 0.0 | 0.0 |
| PL_B4_C_1 | 0.0 | 33.0 | 103.0 | 5.0 | 0.0 | 0.0 | 0.0 | 0.0 | 0.0 | 0.0 | 0.0 | 0.0 | 0.0 | 0.0 | 0.0 |
| PL_B4_C_5 | 283.0 | 0.0 | 0.0 | 0.0 | 392.0 | 0.0 | 0.0 | 10.0 | 0.0 | 0.0 | 0.0 | 0.0 | 0.0 | 0.0 | 0.0 |
| PL_B4_C_6 | 639.0 | 0.0 | 0.0 | 0.0 | 313.0 | 0.0 | 0.0 | 6.0 | 0.0 | 0.0 | 0.0 | 0.0 | 0.0 | 0.0 | 0.0 |
| PL_B4_C_7 | 289.0 | 0.0 | 0.0 | 0.0 | 321.0 | 0.0 | 0.0 | 0.0 | 0.0 | 0.0 | 0.0 | 0.0 | 0.0 | 0.0 | 0.0 |
| PL_B4_C_8 | 311.0 | 0.0 | 0.0 | 0.0 | 102.0 | 0.0 | 0.0 | 0.0 | 0.0 | 0.0 | 0.0 | 0.0 | 0.0 | 0.0 | 0.0 |
| PL_B4_C_9 | 524.0 | 0.0 | 0.0 | 0.0 | 157.0 | 0.0 | 0.0 | 0.0 | 0.0 | 0.0 | 0.0 | 0.0 | 0.0 | 0.0 | 0.0 |
| PL_B4NZ_A_0 | 0.0 | 0.0 | 875.0 | 0.0 | 0.0 | 0.0 | 36.0 | 0.0 | 561.0 | 0.0 | 0.0 | 0.0 | 0.0 | 58.0 | 132.0 |
| PL_B4NZ_A_1 | 43.0 | 0.0 | 1449.0 | 0.0 | 0.0 | 0.0 | 1070.0 | 0.0 | 0.0 | 262.0 | 0.0 | 119.0 | 0.0 | 97.0 | 0.0 |
| PL_B4NZ_A_2 | 0.0 | 0.0 | 1307.0 | 0.0 | 0.0 | 0.0 | 112.0 | 0.0 | 0.0 | 206.0 | 0.0 | 100.0 | 0.0 | 8.0 | 0.0 |
| PL_B4NZ_A_3 | 0.0 | 0.0 | 1848.0 | 0.0 | 0.0 | 0.0 | 825.0 | 0.0 | 0.0 | 109.0 | 0.0 | 138.0 | 0.0 | 0.0 | 0.0 |
| PL_B4NZ_A_4 | 0.0 | 0.0 | 1609.0 | 0.0 | 0.0 | 0.0 | 853.0 | 0.0 | 0.0 | 198.0 | 0.0 | 112.0 | 0.0 | 0.0 | 0.0 |
| PL_B4NZ_A_5 | 0.0 | 0.0 | 1458.0 | 0.0 | 0.0 | 0.0 | 105.0 | 0.0 | 0.0 | 391.0 | 0.0 | 122.0 | 0.0 | 0.0 | 0.0 |
| PL_B4NZ_A_6 | 0.0 | 0.0 | 1565.0 | 0.0 | 0.0 | 0.0 | 148.0 | 6.0 | 0.0 | 0.0 | 0.0 | 126.0 | 0.0 | 0.0 | 0.0 |
| PL_B4NZ_A_7 | 0.0 | 0.0 | 885.0 | 0.0 | 0.0 | 0.0 | 861.0 | 0.0 | 0.0 | 0.0 | 0.0 | 67.0 | 0.0 | 0.0 | 0.0 |
| PL_B4NZ_A_8 | 0.0 | 0.0 | 761.0 | 0.0 | 0.0 | 0.0 | 910.0 | 0.0 | 0.0 | 31.0 | 0.0 | 0.0 | 0.0 | 0.0 | 0.0 |
| PL_B4NZ_B_0 | 248.0 | 124.0 | 1217.0 | 0.0 | 0.0 | 0.0 | 0.0 | 0.0 | 162.0 | 13.0 | 0.0 | 0.0 | 0.0 | 0.0 | 0.0 |
| PL_B4NZ_B_1 | 1870.0 | 38.0 | 27.0 | 6.0 | 60.0 | 0.0 | 0.0 | 0.0 | 0.0 | 93.0 | 0.0 | 0.0 | 0.0 | 0.0 | 0.0 |
| PL_B4NZ_B_2 | 1206.0 | 8.0 | 0.0 | 8.0 | 330.0 | 0.0 | 0.0 | 0.0 | 0.0 | 18.0 | 0.0 | 0.0 | 0.0 | 0.0 | 0.0 |
| PL_B4NZ_B_3 | 1264.0 | 0.0 | 0.0 | 0.0 | 1251.0 | 0.0 | 0.0 | 0.0 | 0.0 | 0.0 | 0.0 | 0.0 | 0.0 | 0.0 | 0.0 |
| PL_B4NZ_B_4 | 1180.0 | 0.0 | 0.0 | 7.0 | 1376.0 | 0.0 | 0.0 | 0.0 | 0.0 | 0.0 | 0.0 | 0.0 | 0.0 | 0.0 | 0.0 |
| PL_B4NZ_B_5 | 959.0 | 0.0 | 0.0 | 3.0 | 780.0 | 0.0 | 0.0 | 0.0 | 0.0 | 0.0 | 0.0 | 0.0 | 0.0 | 0.0 | 0.0 |
| PL_B4NZ_B_6 | 905.0 | 0.0 | 0.0 | 0.0 | 1850.0 | 0.0 | 0.0 | 0.0 | 0.0 | 0.0 | 0.0 | 0.0 | 0.0 | 0.0 | 0.0 |
| PL_B4NZ_B_8 | 1875.0 | 0.0 | 0.0 | 0.0 | 879.0 | 0.0 | 0.0 | 0.0 | 0.0 | 0.0 | 0.0 | 0.0 | 0.0 | 0.0 | 0.0 |
| PL_B4NZ_B_9 | 1389.0 | 0.0 | 0.0 | 0.0 | 1219.0 | 0.0 | 0.0 | 0.0 | 0.0 | 0.0 | 0.0 | 0.0 | 0.0 | 0.0 | 0.0 |
| PL_B4NZ_C_0 | 73.0 | 74.0 | 1428.0 | 4.0 | 0.0 | 0.0 | 0.0 | 14.0 | 614.0 | 0.0 | 40.0 | 0.0 | 144.0 | 0.0 | 0.0 |
| PL_B4NZ_C_1 | 81.0 | 864.0 | 255.0 | 39.0 | 0.0 | 0.0 | 0.0 | 1001.0 | 0.0 | 147.0 | 0.0 | 0.0 | 0.0 | 0.0 | 0.0 |
| PL_B4NZ_C_2 | 750.0 | 362.0 | 23.0 | 132.0 | 13.0 | 0.0 | 0.0 | 1054.0 | 0.0 | 25.0 | 0.0 | 0.0 | 0.0 | 0.0 | 0.0 |
| PL_B4NZ_C_3 | 1036.0 | 30.0 | 17.0 | 57.0 | 0.0 | 2.0 | 0.0 | 893.0 | 0.0 | 0.0 | 0.0 | 0.0 | 0.0 | 0.0 | 0.0 |
| PL_B4NZ_C_4 | 1688.0 | 7.0 | 0.0 | 113.0 | 0.0 | 0.0 | 0.0 | 763.0 | 0.0 | 0.0 | 0.0 | 0.0 | 0.0 | 0.0 | 0.0 |
| PL_B4NZ_C_5 | 1212.0 | 0.0 | 0.0 | 14.0 | 0.0 | 0.0 | 0.0 | 654.0 | 0.0 | 0.0 | 0.0 | 0.0 | 0.0 | 0.0 | 0.0 |
| PL_B4NZ_C_6 | 1728.0 | 0.0 | 0.0 | 17.0 | 0.0 | 0.0 | 0.0 | 1054.0 | 0.0 | 0.0 | 0.0 | 0.0 | 0.0 | 0.0 | 0.0 |
| PL_B4NZ_C_7 | 1028.0 | 0.0 | 0.0 | 34.0 | 0.0 | 266.0 | 0.0 | 1456.0 | 0.0 | 0.0 | 0.0 | 0.0 | 0.0 | 0.0 | 0.0 |
| PL_B4NZ_C_8 | 2129.0 | 0.0 | 0.0 | 7.0 | 0.0 | 352.0 | 0.0 | 575.0 | 0.0 | 0.0 | 0.0 | 0.0 | 0.0 | 0.0 | 0.0 |
| PL_B4NZ_C_9 | 1039.0 | 0.0 | 0.0 | 31.0 | 0.0 | 87.0 | 0.0 | 819.0 | 0.0 | 0.0 | 0.0 | 0.0 | 0.0 | 0.0 | 0.0 |
| PL_B4U_A_0 | 0.0 | 1651.0 | 0.0 | 0.0 | 0.0 | 0.0 | 0.0 | 0.0 | 0.0 | 0.0 | 0.0 | 0.0 | 0.0 | 0.0 | 0.0 |
| PL_B4U_A_1 | 0.0 | 2362.0 | 0.0 | 0.0 | 0.0 | 0.0 | 0.0 | 0.0 | 0.0 | 0.0 | 0.0 | 0.0 | 0.0 | 0.0 | 0.0 |
| PL_B4U_A_2 | 0.0 | 2336.0 | 0.0 | 0.0 | 0.0 | 0.0 | 0.0 | 0.0 | 0.0 | 0.0 | 0.0 | 0.0 | 0.0 | 0.0 | 0.0 |
| PL_B4U_A_3 | 0.0 | 2891.0 | 0.0 | 0.0 | 0.0 | 0.0 | 0.0 | 0.0 | 0.0 | 0.0 | 0.0 | 0.0 | 0.0 | 0.0 | 0.0 |
| PL_B4U_A_4 | 0.0 | 1590.0 | 0.0 | 0.0 | 0.0 | 0.0 | 0.0 | 0.0 | 0.0 | 0.0 | 0.0 | 0.0 | 0.0 | 0.0 | 0.0 |
| PL_B4U_A_5 | 0.0 | 2492.0 | 0.0 | 0.0 | 0.0 | 0.0 | 0.0 | 0.0 | 0.0 | 0.0 | 0.0 | 0.0 | 0.0 | 0.0 | 0.0 |
| PL_B4U_A_6 | 0.0 | 2816.0 | 0.0 | 0.0 | 0.0 | 0.0 | 0.0 | 0.0 | 0.0 | 0.0 | 0.0 | 0.0 | 0.0 | 0.0 | 0.0 |
| PL_B4U_A_7 | 0.0 | 2187.0 | 0.0 | 0.0 | 0.0 | 0.0 | 0.0 | 0.0 | 0.0 | 0.0 | 0.0 | 0.0 | 0.0 | 0.0 | 0.0 |
| PL_B4U_A_8 | 2216.0 | 0.0 | 0.0 | 0.0 | 608.0 | 0.0 | 0.0 | 0.0 | 0.0 | 0.0 | 0.0 | 0.0 | 0.0 | 0.0 | 0.0 |
| PL_B4U_A_9 | 0.0 | 1709.0 | 0.0 | 0.0 | 0.0 | 1176.0 | 0.0 | 0.0 | 0.0 | 0.0 | 0.0 | 0.0 | 0.0 | 0.0 | 0.0 |
| PL_B4U_B_0 | 0.0 | 426.0 | 147.0 | 0.0 | 0.0 | 0.0 | 0.0 | 0.0 | 0.0 | 0.0 | 0.0 | 0.0 | 0.0 | 0.0 | 0.0 |
| PL_B4U_B_2 | 0.0 | 2051.0 | 0.0 | 0.0 | 0.0 | 0.0 | 0.0 | 0.0 | 0.0 | 0.0 | 0.0 | 0.0 | 0.0 | 0.0 | 0.0 |
| PL_B4U_B_3 | 0.0 | 2071.0 | 0.0 | 0.0 | 0.0 | 0.0 | 0.0 | 0.0 | 0.0 | 0.0 | 0.0 | 0.0 | 0.0 | 0.0 | 0.0 |
| PL_B4U_B_4 | 0.0 | 7441.0 | 0.0 | 0.0 | 0.0 | 0.0 | 6.0 | 0.0 | 0.0 | 0.0 | 0.0 | 0.0 | 0.0 | 0.0 | 0.0 |
| PL_B4U_B_6 | 0.0 | 1942.0 | 0.0 | 0.0 | 0.0 | 0.0 | 0.0 | 0.0 | 0.0 | 0.0 | 0.0 | 0.0 | 0.0 | 0.0 | 0.0 |
| PL_B4U_B_7 | 0.0 | 2502.0 | 0.0 | 0.0 | 0.0 | 0.0 | 0.0 | 0.0 | 0.0 | 0.0 | 0.0 | 0.0 | 0.0 | 0.0 | 0.0 |
| PL_B4U_B_8 | 0.0 | 2056.0 | 0.0 | 0.0 | 0.0 | 0.0 | 0.0 | 0.0 | 0.0 | 0.0 | 0.0 | 0.0 | 0.0 | 0.0 | 0.0 |
| PL_B4U_B_9 | 0.0 | 2370.0 | 0.0 | 0.0 | 0.0 | 0.0 | 0.0 | 0.0 | 0.0 | 0.0 | 0.0 | 0.0 | 0.0 | 0.0 | 0.0 |
| PL_B4U_C_0 | 0.0 | 1961.0 | 0.0 | 0.0 | 0.0 | 0.0 | 0.0 | 0.0 | 0.0 | 0.0 | 0.0 | 0.0 | 0.0 | 0.0 | 0.0 |
| PL_B4U_C_2 | 0.0 | 2771.0 | 0.0 | 0.0 | 0.0 | 0.0 | 0.0 | 0.0 | 0.0 | 0.0 | 0.0 | 0.0 | 0.0 | 0.0 | 0.0 |
| PL_B4U_C_3 | 0.0 | 1928.0 | 6.0 | 10.0 | 0.0 | 0.0 | 0.0 | 0.0 | 0.0 | 0.0 | 0.0 | 0.0 | 0.0 | 0.0 | 0.0 |
| PL_B4U_C_4 | 0.0 | 1910.0 | 0.0 | 137.0 | 0.0 | 0.0 | 0.0 | 0.0 | 0.0 | 0.0 | 0.0 | 0.0 | 0.0 | 0.0 | 0.0 |
| PL_B4U_C_5 | 0.0 | 2159.0 | 0.0 | 217.0 | 0.0 | 3.0 | 0.0 | 0.0 | 0.0 | 0.0 | 0.0 | 0.0 | 0.0 | 0.0 | 0.0 |
| PL_B4U_C_6 | 0.0 | 1412.0 | 0.0 | 648.0 | 0.0 | 0.0 | 0.0 | 0.0 | 0.0 | 0.0 | 0.0 | 0.0 | 0.0 | 0.0 | 0.0 |
| PL_B4U_C_7 | 5.0 | 1464.0 | 0.0 | 755.0 | 0.0 | 0.0 | 0.0 | 0.0 | 0.0 | 0.0 | 0.0 | 0.0 | 0.0 | 0.0 | 0.0 |
| PL_B4U_C_8 | 0.0 | 1067.0 | 0.0 | 943.0 | 0.0 | 0.0 | 0.0 | 0.0 | 0.0 | 0.0 | 0.0 | 0.0 | 0.0 | 0.0 | 0.0 |
| PL_B4U_C_9 | 0.0 | 2171.0 | 0.0 | 64.0 | 0.0 | 0.0 | 0.0 | 0.0 | 0.0 | 0.0 | 0.0 | 0.0 | 0.0 | 0.0 | 0.0 |
| PL_B4UNZ_A_0 | 166.0 | 120.0 | 1732.0 | 7.0 | 0.0 | 0.0 | 0.0 | 0.0 | 295.0 | 0.0 | 0.0 | 0.0 | 0.0 | 53.0 | 0.0 |
| PL_B4UNZ_A_2 | 2957.0 | 0.0 | 0.0 | 0.0 | 0.0 | 0.0 | 0.0 | 0.0 | 0.0 | 0.0 | 0.0 | 0.0 | 0.0 | 0.0 | 0.0 |
| PL_B4UNZ_A_3 | 2148.0 | 0.0 | 0.0 | 0.0 | 0.0 | 0.0 | 0.0 | 0.0 | 0.0 | 0.0 | 0.0 | 0.0 | 0.0 | 0.0 | 0.0 |
| PL_B4UNZ_A_4 | 2389.0 | 0.0 | 0.0 | 5.0 | 0.0 | 0.0 | 0.0 | 0.0 | 0.0 | 0.0 | 0.0 | 0.0 | 0.0 | 0.0 | 0.0 |
| PL_B4UNZ_A_5 | 2073.0 | 0.0 | 0.0 | 5.0 | 0.0 | 0.0 | 0.0 | 0.0 | 0.0 | 0.0 | 0.0 | 0.0 | 0.0 | 0.0 | 0.0 |
| PL_B4UNZ_A_6 | 2138.0 | 0.0 | 0.0 | 10.0 | 0.0 | 0.0 | 0.0 | 0.0 | 0.0 | 0.0 | 0.0 | 0.0 | 0.0 | 0.0 | 0.0 |
| PL_B4UNZ_A_7 | 936.0 | 0.0 | 0.0 | 99.0 | 0.0 | 0.0 | 0.0 | 0.0 | 0.0 | 0.0 | 0.0 | 0.0 | 0.0 | 0.0 | 0.0 |
| PL_B4UNZ_A_9 | 1593.0 | 0.0 | 0.0 | 12.0 | 0.0 | 0.0 | 0.0 | 0.0 | 0.0 | 0.0 | 0.0 | 0.0 | 0.0 | 0.0 | 0.0 |
| PL_B4UNZ_B_1 | 2594.0 | 66.0 | 94.0 | 0.0 | 0.0 | 0.0 | 50.0 | 0.0 | 0.0 | 0.0 | 0.0 | 0.0 | 0.0 | 0.0 | 0.0 |
| PL_B4UNZ_B_2 | 2022.0 | 10.0 | 0.0 | 0.0 | 0.0 | 0.0 | 77.0 | 0.0 | 0.0 | 0.0 | 0.0 | 0.0 | 0.0 | 0.0 | 0.0 |
| PL_B4UNZ_B_3 | 2389.0 | 0.0 | 0.0 | 0.0 | 0.0 | 0.0 | 30.0 | 0.0 | 0.0 | 0.0 | 0.0 | 0.0 | 0.0 | 0.0 | 0.0 |
| PL_B4UNZ_B_4 | 2590.0 | 0.0 | 0.0 | 0.0 | 0.0 | 0.0 | 43.0 | 0.0 | 0.0 | 0.0 | 0.0 | 0.0 | 0.0 | 0.0 | 0.0 |
| PL_B4UNZ_B_5 | 2267.0 | 0.0 | 0.0 | 0.0 | 0.0 | 0.0 | 7.0 | 0.0 | 0.0 | 0.0 | 0.0 | 0.0 | 0.0 | 0.0 | 0.0 |
| PL_B4UNZ_B_6 | 2596.0 | 0.0 | 0.0 | 8.0 | 0.0 | 0.0 | 0.0 | 0.0 | 0.0 | 0.0 | 0.0 | 0.0 | 0.0 | 0.0 | 0.0 |
| PL_B4UNZ_B_8 | 2516.0 | 0.0 | 0.0 | 9.0 | 0.0 | 0.0 | 77.0 | 0.0 | 0.0 | 0.0 | 0.0 | 0.0 | 0.0 | 0.0 | 0.0 |
| PL_B4UNZ_B_9 | 1868.0 | 0.0 | 0.0 | 0.0 | 0.0 | 0.0 | 22.0 | 0.0 | 0.0 | 0.0 | 0.0 | 0.0 | 0.0 | 0.0 | 0.0 |
| PL_B4UNZ_C_0 | 101.0 | 325.0 | 731.0 | 0.0 | 0.0 | 0.0 | 0.0 | 0.0 | 507.0 | 19.0 | 0.0 | 0.0 | 0.0 | 0.0 | 0.0 |
| PL_B4UNZ_C_1 | 2169.0 | 45.0 | 12.0 | 28.0 | 47.0 | 0.0 | 0.0 | 0.0 | 0.0 | 7.0 | 0.0 | 0.0 | 0.0 | 0.0 | 0.0 |
| PL_B4UNZ_C_2 | 2363.0 | 0.0 | 0.0 | 29.0 | 136.0 | 0.0 | 0.0 | 0.0 | 0.0 | 0.0 | 0.0 | 0.0 | 0.0 | 0.0 | 0.0 |
| PL_B4UNZ_C_3 | 3260.0 | 0.0 | 0.0 | 80.0 | 1489.0 | 0.0 | 0.0 | 0.0 | 0.0 | 0.0 | 0.0 | 0.0 | 0.0 | 0.0 | 0.0 |
| PL_B4UNZ_C_4 | 1537.0 | 0.0 | 0.0 | 20.0 | 837.0 | 0.0 | 0.0 | 0.0 | 0.0 | 0.0 | 0.0 | 0.0 | 0.0 | 0.0 | 0.0 |
| PL_B4UNZ_C_5 | 1104.0 | 0.0 | 0.0 | 5.0 | 807.0 | 0.0 | 0.0 | 0.0 | 0.0 | 0.0 | 0.0 | 0.0 | 0.0 | 0.0 | 0.0 |
| PL_B4UNZ_C_6 | 2120.0 | 73.0 | 0.0 | 0.0 | 1217.0 | 0.0 | 0.0 | 0.0 | 0.0 | 0.0 | 0.0 | 0.0 | 0.0 | 0.0 | 0.0 |
| PL_B4UNZ_C_7 | 1794.0 | 0.0 | 0.0 | 0.0 | 964.0 | 0.0 | 0.0 | 0.0 | 0.0 | 0.0 | 0.0 | 0.0 | 0.0 | 0.0 | 0.0 |
| PL_B4UNZ_C_8 | 6.0 | 1463.0 | 1193.0 | 0.0 | 0.0 | 0.0 | 0.0 | 0.0 | 0.0 | 329.0 | 654.0 | 0.0 | 0.0 | 0.0 | 0.0 |
| PL_B4UNZ_C_9 | 1450.0 | 0.0 | 0.0 | 0.0 | 1152.0 | 0.0 | 0.0 | 0.0 | 0.0 | 0.0 | 0.0 | 0.0 | 0.0 | 0.0 | 0.0 |Abundance
Supplemental Figure 12. 16s rRNA counts of all plate derived consortium. Consortia are shown the x-axis and counts on the y-axis. As an example, PL_B4_4_2 refers to a liquid consortia, in B4 media, replicate A, week 4. A subset of samples gave poor sequencing results and are not displayed.

## Slide 13
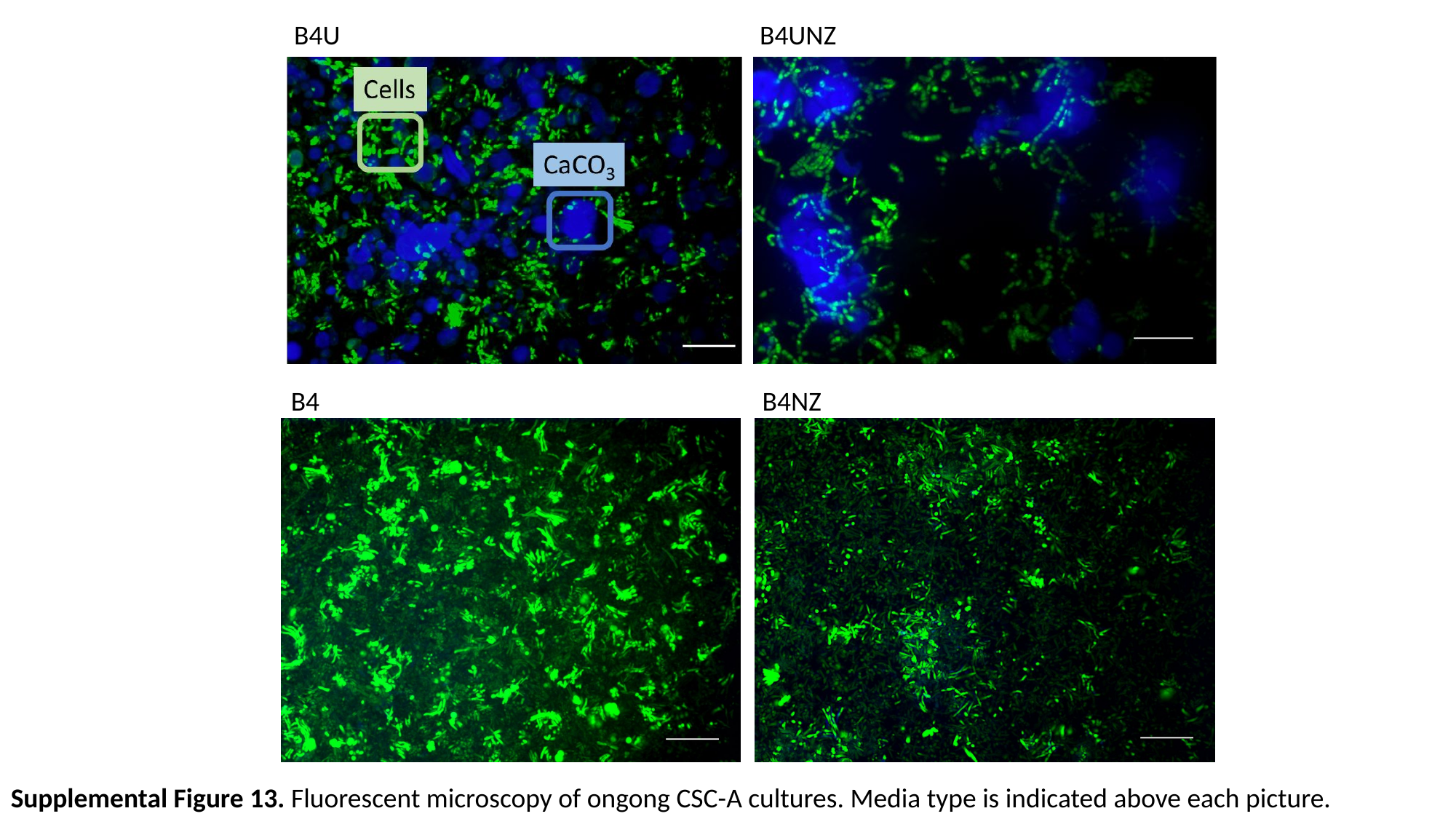

B4U
B4UNZ
B4
B4NZ
Supplemental Figure 13. Fluorescent microscopy of ongong CSC-A cultures. Media type is indicated above each picture. Microbial cells and CaCO3 crystals are indicated in the B4U picture. A 20 uM scale bar is shown in the lower right corner of each picture.

## Slide 14
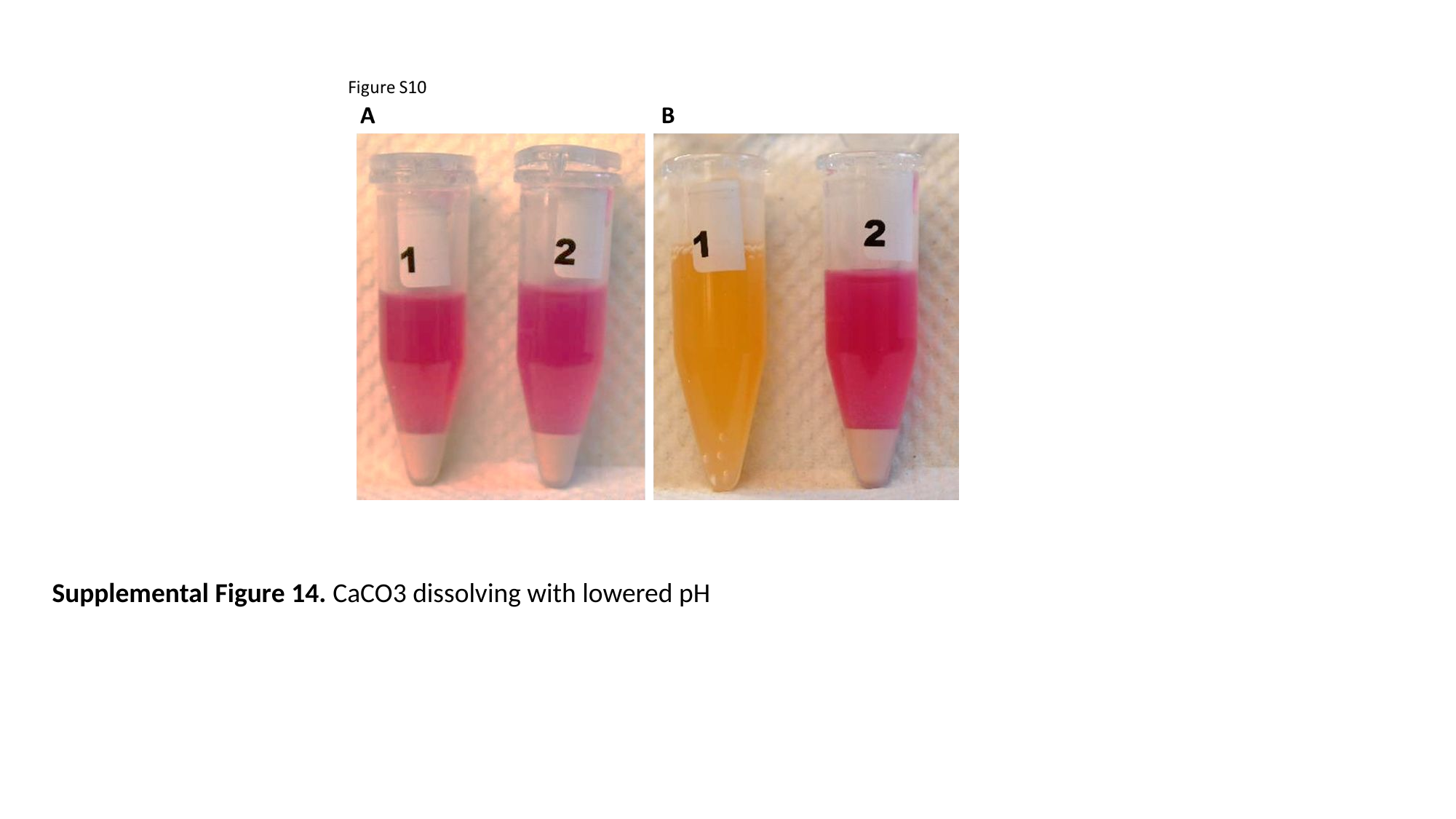

Supplemental Figure 14. CaCO3 dissolving with lowered pH

## Slide 15
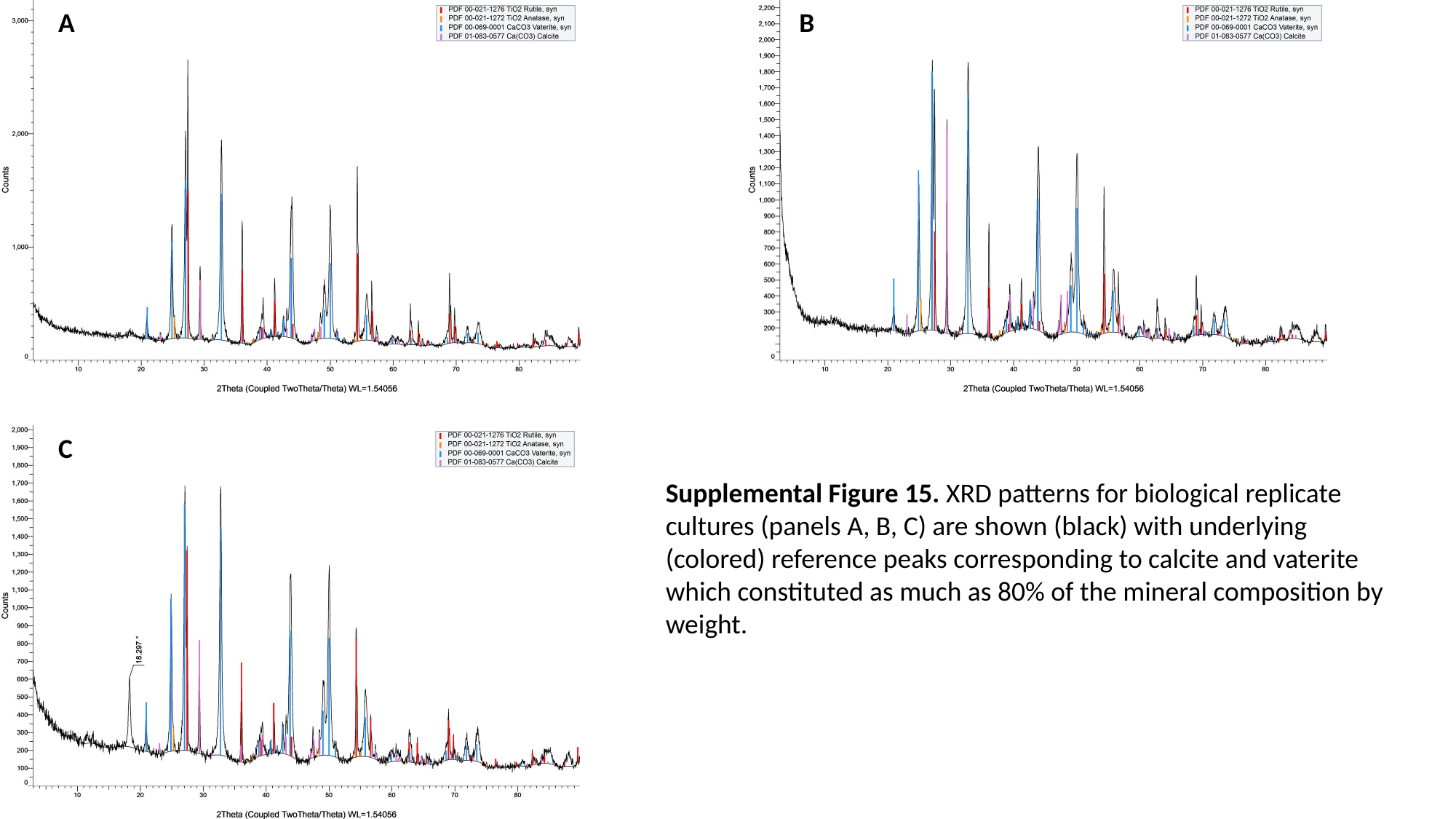

A
B
C
Supplemental Figure 15. XRD patterns for biological replicate cultures (panels A, B, C) are shown (black) with underlying (colored) reference peaks corresponding to calcite and vaterite which constituted as much as 80% of the mineral composition by weight.

## Slide 16
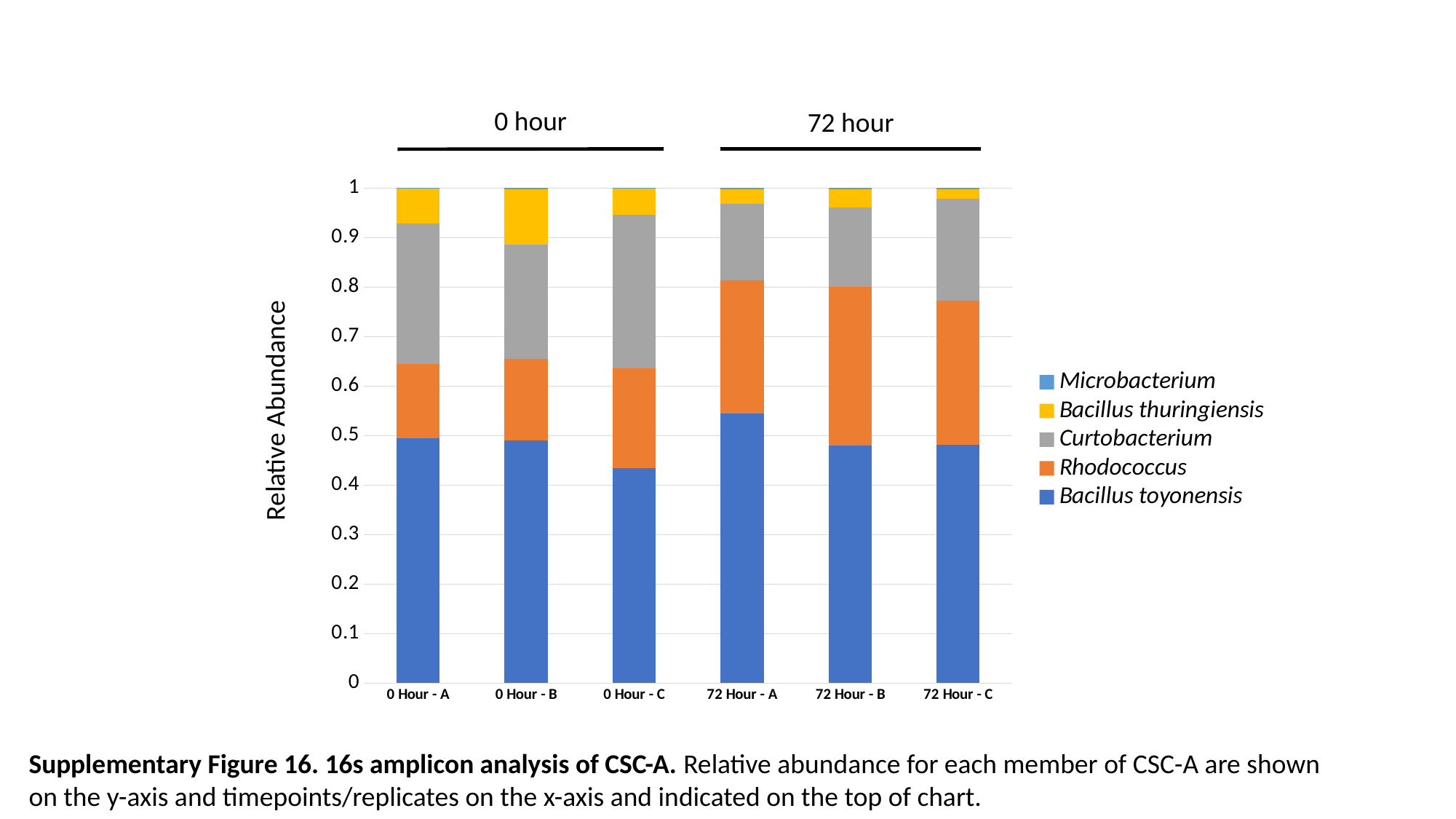

0 hour
72 hour
### Chart
| Category | Bacillus toyonensis | Rhodococcus | Curtobacterium | Bacillus thuringiensis | Microbacterium |
|---|---|---|---|---|---|
| 0 Hour - A | 0.4951712827988338 | 0.15005466472303208 | 0.28316326530612246 | 0.07006195335276968 | 0.0015488338192419825 |
| 0 Hour - B | 0.4899042645778938 | 0.1650130548302872 | 0.2309834638816362 | 0.1122715404699739 | 0.0018276762402088772 |
| 0 Hour - C | 0.4343985867994219 | 0.20202344628231894 | 0.3099405813393287 | 0.05219206680584551 | 0.0014453187730849527 |
| 72 Hour - A | 0.5440836340086964 | 0.26996021833657136 | 0.15339069294106764 | 0.02997502081598668 | 0.002590433897677861 |
| 72 Hour - B | 0.4803854094975912 | 0.32002752924982797 | 0.16035788024776323 | 0.0364762560220234 | 0.0027529249827942187 |
| 72 Hour - C | 0.4812947940458376 | 0.2908561077419863 | 0.20579664487674254 | 0.01953217295424116 | 0.0025202803811924077 |Relative Abundance
Supplementary Figure 16. 16s amplicon analysis of CSC-A. Relative abundance for each member of CSC-A are shown on the y-axis and timepoints/replicates on the x-axis and indicated on the top of chart.
